# Ecd promotes U5 snRNP maturation and Prp8 stability

**DOI:** 10.1093/nar/gkaa1274

**Published:** 2021-01-14

**Authors:** Steffen Erkelenz, Dimitrije Stanković, Juliane Mundorf, Tina Bresser, Ann-Katrin Claudius, Volker Boehm, Niels H Gehring, Mirka Uhlirova

**Affiliations:** Institute for Genetics and Cologne Excellence Cluster on Cellular Stress Responses in Aging-Associated Diseases (CECAD), University of Cologne, Cologne 50931, Germany; Center for Molecular Medicine Cologne (CMMC), University of Cologne, Cologne 50931, Germany; Institute for Genetics and Cologne Excellence Cluster on Cellular Stress Responses in Aging-Associated Diseases (CECAD), University of Cologne, Cologne 50931, Germany; Center for Molecular Medicine Cologne (CMMC), University of Cologne, Cologne 50931, Germany; Institute for Genetics and Cologne Excellence Cluster on Cellular Stress Responses in Aging-Associated Diseases (CECAD), University of Cologne, Cologne 50931, Germany; Institute for Genetics and Cologne Excellence Cluster on Cellular Stress Responses in Aging-Associated Diseases (CECAD), University of Cologne, Cologne 50931, Germany; Institute for Genetics and Cologne Excellence Cluster on Cellular Stress Responses in Aging-Associated Diseases (CECAD), University of Cologne, Cologne 50931, Germany; Center for Molecular Medicine Cologne (CMMC), University of Cologne, Cologne 50931, Germany; Institute for Genetics, University of Cologne, Cologne 50674, Germany; Center for Molecular Medicine Cologne (CMMC), University of Cologne, Cologne 50931, Germany; Institute for Genetics, University of Cologne, Cologne 50674, Germany; Institute for Genetics and Cologne Excellence Cluster on Cellular Stress Responses in Aging-Associated Diseases (CECAD), University of Cologne, Cologne 50931, Germany; Center for Molecular Medicine Cologne (CMMC), University of Cologne, Cologne 50931, Germany

## Abstract

Pre-mRNA splicing catalyzed by the spliceosome represents a critical step in the regulation of gene expression contributing to transcriptome and proteome diversity. The spliceosome consists of five small nuclear ribonucleoprotein particles (snRNPs), the biogenesis of which remains only partially understood. Here we define the evolutionarily conserved protein Ecdysoneless (Ecd) as a critical regulator of U5 snRNP assembly and Prp8 stability. Combining *Drosophila* genetics with proteomic approaches, we demonstrate the Ecd requirement for the maintenance of adult healthspan and lifespan and identify the Sm ring protein SmD3 as a novel interaction partner of Ecd. We show that the predominant task of Ecd is to deliver Prp8 to the emerging U5 snRNPs in the cytoplasm. *Ecd* deficiency, on the other hand, leads to reduced Prp8 protein levels and compromised U5 snRNP biogenesis, causing loss of splicing fidelity and transcriptome integrity. Based on our findings, we propose that Ecd chaperones Prp8 to the forming U5 snRNP allowing completion of the cytoplasmic part of the U5 snRNP biogenesis pathway necessary to meet the cellular demand for functional spliceosomes.

## INTRODUCTION

The defining feature of most eukaryotic genes is the presence of introns, non-coding sequences that interrupt discrete coding genomic units called exons. For genetic information to be realized, the introns have to be excised from nascent pre-mRNAs and the exons precisely fused in the process of pre-mRNA splicing. Importantly, through alternative selection of exons and splice sites during alternative splicing (AS) the coding capacity of genomes markedly increases, thereby enhancing the diversity of the transcriptome and the proteome. In contrast, aberrant splicing events have been recognized as drivers of genetic diseases and implicated in different cancers (1,2), promoting significant interest in an in-depth understanding of the intricate molecular mechanisms that safeguard the fidelity of the splicing process.

Intron excision from pre-mRNAs is catalyzed by a large molecular machine called the spliceosome. The spliceosome consists of five major building blocks, the U1, U2, U4, U5 and U6 small nuclear ribonucleoprotein particles (U snRNPs) and a broad array of proteins that dynamically associate with the complex. Each snRNP contains a short non-coding uridine-rich RNA (*U snRNA*), a common heptameric Sm or Sm-like protein ring, and a specific set of proteins (3,4). The biogenesis of U snRNPs is a stepwise process that starts with the transcription of *U snRNAs* in the nucleus by RNA polymerase II (RNA Pol II) as in the case of *U1*, *U2*, *U4* and *U5 snRNA*, or RNA polymerase III (RNA Pol III) that synthesizes *U6 snRNA*. While *U6 snRNA* is thought to remain in the nucleus (5), RNA Pol II-synthesized *snRNAs* are exported to the cytoplasm, where the survival motor neuron (SMN) complex guides the formation of a heptameric Sm protein ring around a conserved Sm binding motif (3,4,6). The core snRNP particle then serves as a binding platform for a suite of snRNP-specific proteins. While the early SMN-dependent steps of U snRNP assembly are well described, the incorporation of snRNP-specific proteins into the maturing particles are mechanistically far less understood.

Prp8 is among the largest and the most conserved proteins of the spliceosome across taxa (7). As a core component of U5 snRNP, Prp8 is vital for the organization of the catalytic center of the spliceosome by positioning critical snRNAs and substrate pre-mRNA residues, but also for spliceosome activation by coordinating the Brr2/SNRNP200 helicase in cooperation with GTPase Snu114/EFTUD2 (8–12). Prp8 together with Snu114 and Aar2 joins the core U5 snRNP in the cytoplasm, forming a precursor U5 snRNP (13–16). After nuclear import, Aar2 is substituted by Brr2, and extensive snRNA base modifications (17,18) conclude the maturation of functional U5 snRNP. Recent studies implicated the Hsp90 co-chaperone R2TP/Prefoldin-like (R2TP/PFDL) complex in the transfer of pre-formed Prp8 and Snu114/EFTUD2 sub-complexes to assembling U5 snRNPs in the cytoplasm (19). The R2TP/PFDL complex has been described to promote Hsp90-mediated incorporation of client proteins into larger molecular assemblies essential for cell growth and gene expression, including RNA Pol II, complexes of PI3 kinase-like kinases (PIKKs), small nucleolar and nuclear RNPs (20,21).

Recent work performed by us and others provided evidence for a physical interaction between the evolutionarily conserved protein Ecdysoneless (Ecd) and constituents of the U5 snRNP, including Prp8 and Aar2, in *Drosophila* and mammalian cells, pointing towards an involvement of Ecd in pre-mRNA splicing (19,22–24). We further demonstrated that missplicing of the *spok* gene, encoding a key steroidogenic enzyme, contributes to the developmental arrest of *ecd* deficient larvae and their inability to undergo metamorphosis (22). Remarkably, Ecd was also found to interact with subunits of the R2TP/Prefoldin-like (R2TP/PFDL) co-chaperone complex (23,25,26), suggesting that Ecd might act as a molecular linker that bridges the R2TP complex to the U5 snRNP for biogenesis. Yet, its mechanistic engagement with the splicing machinery remains unknown. Importantly, *ecd* is an essential gene required for cell survival during development in *Drosophila* and mammals (22,25,27,28). Intriguingly, elevated Ecd levels were observed in pancreatic (29) and gastric cancers (30) and positively correlated with faster progression and poor prognosis in breast cancer patients (31).

Here, we demonstrate that Ecd is not only vital during development but also indispensable for the maintenance of tissue function during adulthood. We identify the heptameric Sm ring protein SmD3 as a novel binding partner of Ecd in *Drosophila* and demonstrate the necessity of SmD3-Ecd interaction for the biogenesis of nascent U5 snRNPs required for maintenance of transcriptome integrity and splicing accuracy.

## MATERIALS AND METHODS

### *Drosophila melanogaster* lines

The following *Drosophila melanogaster* strains were used: *w^1118^* (BDSC; RRID:BDSC_3605), *ecd^1^* (32), *ecd^Δ^* (this study), *w;; FRT2A* (BDSC; RRID:BDSC_1997), *w;; FRT82B* (BDSC; RRID:BDSC_2035), *w; nubbin-Gal4, UAS-myr-mRFP* (*nub>mRFP*, this study), *w; escargot-Gal4, UAS-GFP, tub-Gal80^TS^* (*esg^TS^>*, 33), *w; nubbin-Gal4, UAS-myr-mRFP; tub-Gal80^TS^* (*nub^TS^>mRFP*, this study), *w;; ecd^l(^^3^^)23^ FRT2A* (22), *w;; ecd^Δ^ FRT2A* (recombined in this study), *w; UAS-ecd^RNAi^* (22), *w; UAS-prp8^RNAi^* (VDRC; ID18565), *w;; UAS-Prp8*^*wt*^ (34)*, w;; UAS-SmD3::3xHA* (FlyORF; F003987), *w;; UAS*-*SmD3::3xHA, UAS-ecd^RNAi^* (recombined in this study)*, w; nubbin-Gal4, UAS-myr-mRFP; UAS*-*SmD3::3xHA, tub-Gal80^TS^* (this study)*, w; nubbin-Gal4, UAS-myr-mRFP; ecd^1^* (this study), *w;; UAS*-*SmD3::3xHA, UAS-Prp8*^*wt*^ (recombined in this study), *w;; UAS*-*SmD3::3xHA, UAS-ecd^RNAi^, UAS-Prp8*^*wt*^ (recombined in this study), *eyFLP; act>y^+^>Gal4, UAS-GFP; FRT82B, tub-Gal80* (35), *eyFLP; act>y^+^>Gal4, UAS-GFP; tub-Gal80, FRT2A* (this study), *w; UAS-Ecd^wt^; FRT82B* (this study)*, w; UAS-Myc::Ecd^TripleA^; FRT82B* (this study), *w; UAS-Myc::Ecd^Δ^^34^; FRT82B* (this study), *w; UAS-Ecd^wt^; ecd^Δ^ FRT2A* (this study), *w; UAS-Myc::Ecd^TripleA^; ecd^Δ^ FRT2A* (this study), *w;UAS-Myc::Ecd^Δ^^34^; ecd^Δ^ FRT2A* (this study). Details on *Drosophila* strains are described in Supplementary Table S1.

### Fly Husbandry

*Drosophila* stocks and experimental crosses were kept on a diet consisting of 0.8% agar, 8% cornmeal, 1% soymeal, 1.8% dry yeast, 8% malt extract and 2.2% sugar-beet syrup, supplemented with 0.625% propionic acid and 0.15% Nipagin. Gal4 driver line and *eyFLP MARCM* line crosses were set up and maintained at 25°C unless specified otherwise using *w^1118^* and *FRT2A* or *FRT82B* lines as controls, respectively. For experiments with adult flies, temperature-sensitive *ecd^1^* and control *w^1118^* lines were allowed to develop at 18°C. Crosses using *esg^TS^*> or *nub^TS^>mRFP* expression system were set up and maintained at room temperature. For experiments utilizing *nub^TS^>mRFP*, vials with third instar larvae were upshifted to 29°C for 24 h. For experiments utilizing *esg^TS^>*, eclosing adult flies were collected for 2 days and mated for another 2 days at room temperature before being transferred into fly incubator with a 16 h/8 h light/dark cycle set to 29°C until dissection at indicated age.

### Lifespan experiments

For lifespan experiments *ecd^1^* line was backcrossed to *w*^*1118*^ genetic background for five generations. Mated *ecd^1^* and control *w^1118^* male and female adult flies were separated and transferred into 1 l cages (100 flies/cage) with access to a vial containing the standard fly diet. Cages were placed into fly incubator with a 16 h/8 h light/dark cycle set to 29°C. Dead flies were counted and the food vial was exchanged every 2–3 days. Adult fly lifespan was analyzed in Microsoft Excel (RRID:SCR_016137) and statistical significance was calculated with a log-rank test. The curves prepared with Prism 8 (GraphPad, RRID:SCR_002798) represent one of the two independent experiments. The total number of flies per experiment (*n*), genotype, and condition is specified in the respective figure and its legend.

### Generation of *ecd*^Δ^ loss-of-function allele

The *ecd*^*Δ*^ mutant line was generated using CRISPR–Cas9 genome editing technology utilizing tRNA–sgRNA system (36). pCFD5 plasmid (36; RRID:Addgene_73914) expressing two guide RNAs targeting the exon 1 and 3′ terminus of exon 2 of the endogenous *ecd* gene locus were cloned using the Gibson Assembly reaction (New England Biolabs) according to the manufacturer's instructions with purified PCR fragments amplified with Phusion HS II polymerase (ThermoFisher Scientific Cat# F549L). The *pCFD5-gRNA-Ecd* construct was integrated into the *attP40* site on the second chromosome (25C6) using PhiC31 integrase-mediated transgenesis (BDSC; RRID:BDSC_79604). The resulting stock *v; pCFD5-gRNA-Ecd attP40* was crossed to *nos-cas9, Lig4^169^* fly line (this study). The offspring were collected, balanced and screened for the presence of the deletion via PCR. The recovered *ecd*^Δ^ allele encodes 40 amino acid long peptide of which the first 23 amino acids align to the wild-type Ecd protein while the 17 remaining are unrelated due to a frameshift (Supplementary Figure S1A). sgRNA and primer sequences are presented in Supplementary Table S2.

### Generation of plasmids and transgenic lines

*Drosophila melanogaster* coding DNA sequences of PIH1D1 (CG5792), Pontin (CG4003), SmD3 (CG8427), SmB (CG5352), SmG (CG9742), SmF (CG16792) and Ecd (CG5714) were amplified from respective cDNAs with Phusion HS II polymerase (ThermoFisher Scientific Cat# F549L) and cloned into the pENTR4 dual selection vector (ThermoFisher Scientific Cat# A10465). Ecd^[E496A, E566A, E621A]^ (Ecd^TripleA^) variant was generated by combining a site-directed mutagenesis with an overlap extension PCR. The Ecd^Δ34^ truncated variant was produced by PCR resulting in an exchange of Q650 to a premature stop codon. The N-terminal Myc-, or Flag-tag was added by LR Clonase II-mediated recombination (Invitrogen Cat# 11791020) into the pTMW or pTFW vector (DGRC Cat# 1107 or Cat# 1115). Following reamplification and restriction with EcoRI and KpnI or NotI the Myc::Ecd cassettes were inserted into the pUAST attB vector backbone (DGRC Cat# 1419, 37). All primers and plasmids are listed in Supplementary Table S2.

Transgenic fly lines allowing overexpression of wild-type and mutant Ecd variants were established either by a standard P-element-mediated transformation (Myc::Ecd^Δ34^) into *w^1118^* stock (BDSC; RRID:BDSC_3605) or PhiC31 integrase-mediated transgenesis into *attP40* site (BDSC; RRID:BDSC_25709) (Ecd^wt^, Myc::Ecd^TripleA^).

### Generation of EAD genetic mosaics

The Mosaic Analysis with a Repressible Cell Marker (MARCM) technique (38) with *eyFLP; act>y^+^>Gal4, UAS-GFP; tub-Gal80*, *FRT2A* and *eyFLP; act>y^+^>Gal4, UAS-GFP; FRT82B, tub-Gal80* flies was used to generate genetically defined clones within the EADs as described in (39).

### *Drosophila* S2 cell culture

*Drosophila* Schneider 2 (S2) cells were cultured at 25°C in Shields and Sang M3 insect medium (Sigma-Aldrich Cat# S8398) containing 8% fetal bovine serum (Gibco, Life Technologies) without antibiotics. Cells were transfected in six-well plates (5 × 10^5^ cells/well) in serum-free medium using Trans-IT-Insect reagent (Mirus Bio LLC, MIR6100) following the manufacturer's instructions. Expression of UAS-driven cDNAs was induced by co-transfection with a pWA-GAL4 plasmid expressing Gal4 under an *actin5C* promoter. pIE-EGFP plasmid (40) was co-transfected to monitor transfection efficiency. Ecd knockdown was achieved by transfection of 1.5 μg of purified *in vitro* transcribed double-stranded RNA directed against the 67 nucleotides long 3′ untranslated region (3′ UTR) of the *ecd* mRNA. A dsRNA targeting the *LacZ* gene served as a mock control. Cells were processed for downstream applications 72 h after transfection. To inhibit autophagy-lysosomal pathway, S2 cells were treated for 24 h with 100 μM of Chloroquine (Sigma-Aldrich, Cat# C6628) or 400 nM Bafilomycin A1 (Enzo Life Sciences, Cat# BML-CM110-0100) prior to cell lysis.

### Tissue dissection and immunostaining

EADs and wing imaginal discs dissected from third instar *Drosophila* larvae (7 days AEL) were fixed for 25 min with 4% paraformaldehyde in PBS containing 0.1% Triton X (PBS-T) at room temperature. Adult female intestines were fixed with 4% paraformaldehyde in PBS for 48 h at 4°C. Fixed tissues were washed three times with PBS-T, intestines in PBS only. Primary antibodies were diluted in blocking buffer (PBS-T with 0.3% BSA) and tissues were stained overnight at 4°C. The following primary antibodies were used: rabbit anti-*Drosophila* cleaved Death caspase 1 (Dcp-1, 1:500, Cell Signaling Technology Cat #9578, RRID:AB_2721060), rat-anti Ecd N-term (1:500, 22), mouse-anti Armadillo (Arm, 1:20, Developmental Studies Hybridoma Bank #N2 7A1; RRID:AB_528089), mouse-anti disc large (Dlg1, 1:100, Developmental Studies Hybridoma Bank #4F3; RRID:AB_528203). After washing, the samples were incubated with the corresponding Cy3- or Cy5-conjugated secondary antibodies (1:1000, Jackson ImmunoResearch Labs Cat# 711-175-152, RRID:AB_2340607, Cat# 712-165-150, RRID:AB_2340666, Cat# 715-165-151, RRID:AB_2315777) for 2 h at room temperature and counterstained with DAPI (1:1000 dilution of 5 mg/ml stock, Carl Roth GmbH Cat# 6335.1) to visualize nuclei. Tissues were mounted on glass slides in Dabco-Mowiol 4–88 (Sigma-Aldrich Cat# D2522 and Cat# 81381) and imaged within 72 h.

### Image acquisition and processing

Tissues were imaged on an Olympus FV-1000 confocal microscope equipped with 20x UPlan S-Apo (NA 0.85), 40x UPlan FL (NA 1.30) and 60× UPlanApo (NA 1.35) objectives. Images of the intestines were always taken from the R5 posterior midgut region. All micrographs show maximum projections (unless otherwise indicated) generated with FluoView FV-10ASW software (Olympus, RRID:SCR_014215). Final image processing including panel assembly, brightness and contrast adjustments were performed in Adobe Photoshop CC (Adobe Systems, Inc., RRID:SCR_014199). White outlines of the EADs were drawn based on DAPI staining.

To measure the midgut diameter, bright field images of female adult midguts were taken with the cellSens Standard 1.11 software using an Olympus CKX41 inverted microscope and imported into Adobe Photoshop CC (Adobe Systems, Inc., RRID:SCR_014199). Measurements were taken at a distance of 200 μm anterior to the midgut-hindgut boundary for each gut from outside the visceral mesoderm surrounding the midgut. Statistical significance was determined in GraphPad Prism (GraphPad, RRID:SCR_002798) using an unpaired two-tailed Student's *t*-test with unequal variance.

The number of cells in the imaged R5 posterior midgut region of female adult flies of indicated genotypes was determine based on nuclear DAPI staining from maximum intensity Z-projected confocal stacks using a customized pipeline in CellProfiler (41, RRID:SCR_007358). Different cell types were manually counted in Fiji (RRID:SCR_002285). The progenitors (ISCs and EBs) and EEs were distinguished from large absorptive ECs based on their morphology, smaller nuclear and cell size (DAPI and Dlg1 signal, respectively) and enrichment of an Arm signal. To quantify apoptosis, the Dcp-1 fluorescent signal was thresholded for each replicate and overlayed with DAPI, Arm and Dlg1 channel to facilitate manual counting of Dcp-1-positive ECs and progenitor and EEs in Fiji (RRID:SCR_002285). Statistical significance was determined in GraphPad Prism (GraphPad, RRID:SCR_002798) using unpaired nonparametric two-tailed Mann-Whitney test.

For quantification of the GFP-positive clonal volume, confocal Z-stacks of mosaic EADs were imported into Fiji (RRID:SCR_002285). After thresholding, the individual slices were converted to binary images and the outlines selected. The ratio of GFP and DAPI was determined using the 3D manager plug-in. Same macros were applied to all samples. Macros used are available upon request. Statistical significance was determined in GraphPad Prism (GraphPad, RRID:SCR_002798) using ordinary one-way ANOVA with Tukey's multiple comparisons test.

Brightfield and fluorescent images of female mosaic adult eyes were taken within 24 h after eclosion using an Olympus SZX16 fluorescent stereomicroscope equipped with a DP72 CCD camera under the same magnification and settings. Images were processed with Olympus cellSens 1.1 Software (Olympus, RRID:SCR_014551).

### Preparation of samples for western blotting and immunoprecipitations

Wing discs (15–20 WDs/replicate) or eye-antennal imaginal discs (20–30 EADs/replicate) were dissected from third instar larvae in 1× PBS, and immediately lysed in 50 mM Tris–HCl (pH 7.8), 150 mM NaCl, 1 mM EDTA (pH 8.0), 1% Triton X-100, 0.01% Igepal and protease inhibitors (Roche Applied Science Cat #11 873 580 001). Three to five adult flies were squished and lysed using the same buffer. *Drosophila* S2 cells were pelleted (800 × g at 4°C for 10 min) and resuspended in the same lysis buffer. All samples were cleared of debris and DNA by centrifugation (12 000 × g at 4°C for 10 min). Protein concentration was determined by Bradford assay (Roth GmbH Cat# K015.1) according to manufacturer's instructions.

For immunoprecipitation assays, 250–1000 μg of total protein extracted from S2 cells or third instar larval imaginal discs were incubated overnight at 4°C with the anti-Myc-tag (MBL International Cat# M047-11B), anti-Flag-tag (Sigma-Aldrich Cat# M8823, RRID:AB_2637089) mAb-Magnetic Beads or protein G Dynabeads (Invitrogen Cat# 10004D) coupled to the desired primary antibody. After five washes in lysis buffer containing 150–250 mM NaCl, bound proteins were eluted with 0.1 M glycine–HCl (pH 3.0) for 5 min followed by neutralization with 0.5 M Tris–HCl (pH 7.8) and 1.5 M NaCl.

For western blotting, samples were denatured by boiling in Laemmli buffer containing 2.5% β-mercaptoethanol for 5 min at 95°C. Samples were resolved on 10% or 12% SDS-PAGE. Candidate proteins were detected by immunoblotting with primary antibodies: rabbit anti-dPrp8-CTD (1:1000, 34), rat-anti Ecd N-term (1:1000, 22), mouse anti-Flag M2 (1:1000, Sigma-Aldrich Cat# F1804, RRID:AB_262044), rabbit anti-Myc (1:2000, Cell Signaling Technology Cat# 2272, RRID:AB_10692100), rabbit anti-HA (1:2000, Abcam Cat# ab9110, RRID:AB_307019), mouse anti-ATP5α (1:2000, Abcam Cat# ab14748, RRID:AB_301447), rabbit anti-RFP (1:1000, MBL Cat# PM005, RRID:AB_591279), rabbit anti-GFP (1:2000, Acris Cat# TP401, RRID:AB_10013661) followed by incubation with corresponding goat anti-mouse (1:5000, Jackson ImmunoResearch Labs Cat# 715-035-150, RRID:AB_2340770), rabbit (1:5000, Jackson ImmunoResearch Labs Cat# 711-035-152, RRID:AB_10015282) and rat (1:5000, Jackson ImmunoResearch Labs Cat# 712-035-153, RRID:AB_2340639) HRP-conjugated secondary antibodies. Chemiluminescent signal was captured using ImageQuant LAS4000 reader (GE Healthcare, RRID:SCR_014246). Signal intensities were quantified using the ImageJ (RRID:SCR_003070) and normalized to the mean band intensity of the protein of interest for each biological replicate. Unpaired or paired two-tailed Student's *t*-test or ordinary or two-way ANOVA with a post hoc Tukey's multiple comparisons test were used to determine statistical significance for changes in protein levels and binding.

### Recombinant protein expression and purification

To produce His-tagged Ecd and SmD3::HA proteins, respective cDNAs were cloned into pET28b (Sigma Cat# 69865) and Gateway pDEST17 (Invitrogen Cat# 11803012) vector, respectively. Recombinant proteins were expressed in BL21 Rosetta (DE3) *Escherichia coli* cells (Invitrogen Cat# C600003) upon induction with 0.4 mM IPTG (Sigma-Aldrich Cat# I5502-5G) overnight at 16°C and subsequently affinity purified using Ni Sepharose (GE Healthcare Cat# GE17-5268-01). For pull-down assays, 10 μg of purified His::Ecd and His::SmD3::HA protein were incubated either alone or together overnight at 4°C with protein G Dynabeads (Invitrogen Cat# 10004D) coupled to 3 μg of rat-anti Ecd N-term (22) antibody. Washing steps and subsequent detection via western blotting was carried out as described above.

### Mass spectrometry and protein identification

For proteomic analysis, IP samples (four biological replicates/condition) were washed additional three times in a washing buffer 50 mM Tris–HCl (pH 7.8), 150–250 mM NaCl, 1 mM EDTA (pH 8.0), and protease inhibitors (Roche Applied Science Cat #11 873 580 001) without detergents. Bound proteins were eluted from the beads using 8M urea (Sigma Cat# U1250). For reduction and alkylation of the disulfide bonds, samples were incubated for 30 min at room temperature with 10 mM DTT (Applichem Cat# 3483-12-3) and 20 min with 55 mM iodoacetamide (Merck Cat# 8.04744.0025) in the dark. Proteins were digested with LysC (Wako Cat# 129-02541) for 2–3 h and Trypsin (Serva Cat# 9002-07-7) overnight. Digestion was stopped by addition of 1% formic acid (Honeywell Fluka Cat# 607-001-00-0). Finally, peptides were purified using styrene divinyl benzene (SDB) stage tips (Affinisep, SPE-Disk-Bio-RPS-47.20) and loaded onto the mass spectrometer. For LC–MS/MS analysis, an easy nLC 1000 (ThermoFisher Scientific) was coupled to the quadrupole based QExactive Plus Orbitrap (ThermoFisher Scientific) instrument by a nano-spray ionization source.

All raw files were processed with Maxquant (version 1.5.3.8) (42) and the Andromeda search engine (43). The recorded MS2 spectra were compared to the *Drosophila* reference proteome database (Uniprot Release 2017). Default mass tolerance and modification settings were used. The minimal peptide length was set to seven amino acids and N-terminal acetylation, as well as oxidation at methionine residues, were defined as variable modifications. Cystein-carbamidomethylation was set as a fixed modification. False discovery rates (FDRs) on protein and peptide spectrum match (PSM) level were computed by the target-decoy approach to 1% (44). The iBAQ quantification approach and match-between-runs option were enabled. LFQ intensities were log_2_ transformed and used in a two-sided t-test assuming equal variances to identify significant differently pulled down proteins. Proteomics data were visualized using InstantClue (http://www.instantclue.uni-koeln.de) and the Cytoscape (RRID:SCR_003032). The GO enrichment analysis of immunoprecipitated proteins (log_2_ Difference ≥ 2, *P*-value < 0.05) was performed using the Panther (Protein Analysis THrough Evolutionary Relationships) classification system (www.geneontology.org). Only GO terms with a fold enrichment ≥ 15 (SmD3::HA interactome) and ≥ 5 (Myc::Ecd^wt^ interactome and comparative SmD3::HA versus SmD3::HA, ecd^RNAi^ interactome) and an adjusted *P*-value < 0.05 were taken into account. For visualization, GO terms were clustered with the help of REViGO (RRID:SCR_005825) (45) to remove redundancies using medium (0.7) or small (0.5) setting.

### RNA isolation, RNA immunoprecipitation and Gene expression analysis

Total RNA was isolated from eight third instar larvae or 70–90 WDs using the standard protocol with TRI Reagent (Sigma Aldrich Cat# T9424) and DNase I (Invitrogen Cat# AM2238) treatment (39). For RNA immunoprecipitation (RIP), 450 μg of total protein extracted from third instar larval wing imaginal discs was incubated overnight at 4°C with protein G Dynabeads (Invitrogen Cat# 10004D) conjugated to 2 μg of rabbit anti-HA (1:2000, Abcam Cat# ab9110, RRID:AB_307019) or 3 μg of rat anti-Ecd N-term (22) antibody. After five washes in lysis buffer containing 150 mM NaCl, 1/4 of the bound fraction was subjected to the protein elution protocol as described above to control for equal precipitation efficiencies, while the rest was eluted with TRI Reagent (Sigma Aldrich, Cat# T9424). cDNA was synthesized from 1 to 2 μg of RNA using random hexamer primers and Superscript III Reverse Transcriptase (Invitrogen Cat# 18080044). Semi-quantitative PCR (semi-qPCR) products were separated on 1.5–3% agarose gels and stained with ethidium bromide for visualization. Separate PCR reactions detecting *rp49* mRNA levels (semi-qPCR) and *U5 snRNA* levels within the input samples (RIP) were carried out to control for variations in sample preparations. Quantitative PCR (qPCR) was performed in triplicates with the GoTaq qPCR Master Mix (Promega Cat# A600A) using the CFX96 (Bio-Rad) real-time PCR system. Data were normalized to the levels of *rp49* transcript and fold changes were calculated using the ΔΔCT method (46). An unpaired two-tailed Student's *t*-test with a post hoc Welch's correction or two-way ANOVA with a post hoc Tukey's multiple comparison test were used to determine statistical significance for changes in gene expression (qPCR) or *U snRNA* binding (RIP). All primer sequences are listed in Supplementary Table S2.

### mRNA-seq, transcriptome and splicing analysis

Total RNA was isolated as described above from *w^1118^* and *ecd*^*1*^ larvae that were reared at 18°C for 10 days and upshifted to 29°C for 2 days in biological triplicates. RNA libraries were generated according to the Illumina protocol and pair-end sequenced on an Illumina HiSeq 2000 instrument at 100 bp read length. Image analysis and base calling were carried out with the Illumina RTA software at run time. Initial quality check of the raw data was performed using FastQC (RRID:SCR_014583) (Supplementary Dataset S3), and the reads were aligned to the *Drosophila* reference genome BDGP Release 6 (dm6) using Tophat2 v2.0.10 (http://ccb.jhu.edu/software/tophat) (47). The mapped reads summarization was performed on the gene meta-feature level using featureCounts v1.6.4 (RRID:SCR_012919) (48). Differential gene expression was assessed using DESeq2 (RRID:SCR_015687) (49). Genes satisfying the conditions of log_2_ fold-change, |log_2_ FC| ≥ 1 and adjusted *P*-value < 0.05 were considered as differentially expressed compared to the control samples. The GO term enrichment analysis was performed using GOrilla (http://cbl-gorilla.cs.technion.ac.il) (RRID:SCR_006848) (50), with a custom background gene list comprising of all genes identified by the analysis (Supplementary Dataset S3). GO term clustering was performed using REViGO (RRID:SCR_005825) (45). Heatmaps of log2(x+1) transformed normalized counts for selected genes were generated using the heatmap2 v3.0.1 of the R gplots package and the data for each row are shown as *Z*-scores. Differential splicing was detected with *LeafCutter* (version 0.2.8) (51) using the Tophat2 mapped reads, the dm6 annotation and the parameters min_samples_per_intron = 3 and min_samples_per_group = 3. Significance thresholds were |deltaPSI| > 0.1 and adjusted *P*-value < 0.05. Simple classification of alternative splicing events was performed using a custom script based on the bedtools intersect command (version 2.29.0; with options -s -wao) (52) to extract the exon coordinates from the dm6 annotation overlapping the respective junction, followed by calculating and classifying the distances of each exon to the respective junction (Supplementary Dataset S4). To minimize erroneous classifications due to annotated, but not expressed transcripts/exons, the classification was repeated using a merged *de novo* assembled transcriptome with StringTie (version 1.3.4d; isoform threshold of 0.1) (53). Only classifications that matched both dm6 and the StringTie approaches were selected for final analyses and UpSet plots (54).

## RESULTS

### Ecd is required to maintain adult tissue function

**Figure 1.**
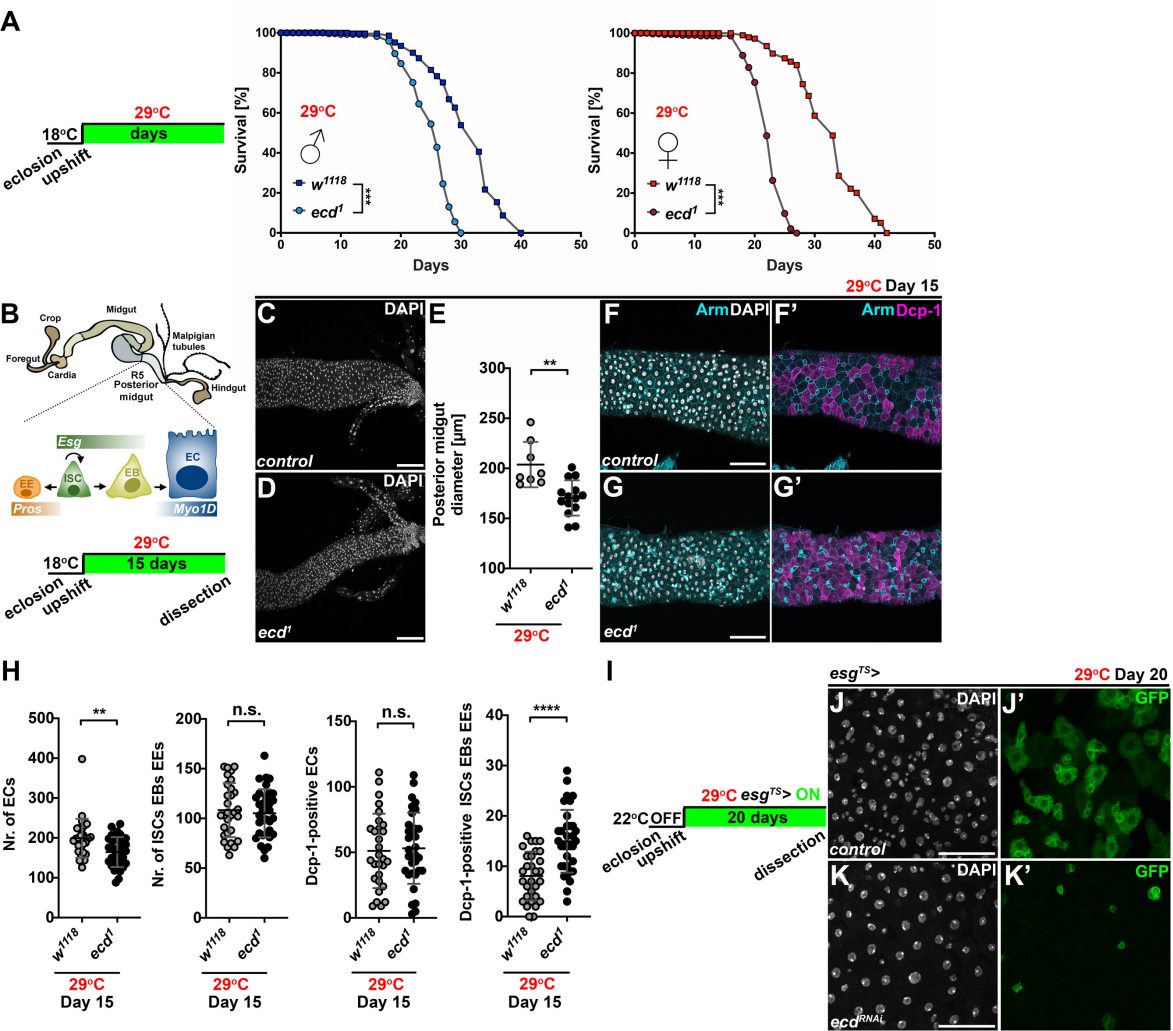
Loss of *ecd* shortens lifespan and compromises maintenance of the adult intestine. (**A**) Control *w*^*1118*^ and temperature-sensitive *ecd^1^* fly lines were allowed to develop at 18°C. After eclosion and two-day mating, males and females were separated and upshifted to restrictive temperature of 29°C. Lifespan curves show percentage of survival of *w^1118^* and homozygous *ecd^1^* adult males and females over time (*n* = 300) at 29°C and represent one of the two independent experiments. Adult *ecd^1^* male and female flies were shorter lived (mean difference of 6.1 days for males and 10.2 days for females) compared to control flies. Statistical significance was determined by a log-rank test, ***P < 0.001. (**B**) Schematic diagram of *Drosophila* adult intestine. Posterior midgut is maintained by the intestinal stem cells (ISCs), which divide asymmetrically to self-renew and generate either the postmitotic enteroblasts (EBs) or the enteroendocrine (EE) lineage precursors. The midgut ISCs and EBs express the transcription factor Escargot (Esg) while EEs are positive for Prospero (Pros). EBs further differentiate into large absorptive enterocytes (ECs) characterized by polyploid nuclei and expression of Myosin 1A (Myo1A). Schematics of experimental setup and timeline to assess impact of *ecd* deficiency on adult midgut. (**C**, **D**) Representative confocal micrographs show that posterior midguts of *ecd^1^* homozygous mutant flies (D) kept at restrictive temperature for 15 days are thinner compared to controls (*w^1118^*) (C). Scale bars: 50 μm. (**E**) Diameter measurements of posterior midguts from control (*w^1118^*, *n* = 8) and *ecd^1^* flies (*n* = 12) kept at 29°C for 15 days. Nuclei were stained with DAPI. Statistical significance was determined by unpaired two-tailed Student's *t*-test. Data represent means ± SD, **P < 0.01. (**F**, **G**) Representative confocal micrographs of posterior midguts from adult female flies kept at restrictive temperature for fifteen days show increased number of apoptotic Dcp-1-positive cells (magenta) in *ecd^1^* (G, G’) compared to control (*w^1118^*) intestines (F, F’). Cell membranes were visualized by Armadillo (Arm) antibody (cyan). Note the stronger Arm signal highlighting smaller progenitors compared to weaker staining of large polyploid ECs (F’, G’). DAPI stains nuclei. Scale bars: 50 μm. (**H**) Cell counts and quantification of Dcp-1-positive cells revealed reduced number of ECs and elevated apoptosis in progenitor and EE population in posterior midguts of *ecd^1^* adult female flies (*n* = 34) kept at 29°C for 15 days compared to control (*w^1118^*, *n* = 30). Unpaired nonparametric two-tailed Mann–Whitney test was used to calculate *P*-values. Data represent means ± SD, **P < 0.01, ****P < 0.0001, n.s. = non-significant. (**I**) Experimental setup and timeline for RNAi-mediated silencing of *ecd* in progenitors (ISCs and EBs) of adult midguts using *esg*^TS^*>* expression system. (**J**, **K**) Representative confocal images of twenty-day-old control posterior midguts (J) and those expressing *ecd^RNAi^* (K) in ISCs/EBs (*esg^TS^>*) marked by GFP show decrease in Esg-positive progenitor cells following *ecd* knockdown (compare J’ and K’). DAPI stains nuclei. Scale bars: 50 μm. See also Supplementary Figure S1.

The Ecd interactions with the core splicing factors indicate that it plays a more general role in pre-mRNA splicing, which is required not only during development but also adulthood. To test the necessity of Ecd function during adulthood, we took advantage of the temperature sensitive, recessive *ecd^1^* mutant allele that carries a single proline-656 to serine (P656S) substitution (Supplementary Figure S1A). While *ecd^1^* homozygous mutant animals develop into viable and fertile adults when kept at permissive temperature (18–22°C), the Ecd function can be abolished by shifting to a restrictive temperature of 29°C (27,32 and Supplementary Figure S1B-E). Intriguingly, when adult flies were upshifted to and maintained at 29°C from day 3 after eclosion, *ecd^1^* homozygous males as well as females lived significantly shorter compared to *w^1118^* controls (Figure 1A). The integrity and function of *Drosophila* adult gastrointestinal tract have emerged as important determinants of animal health and lifespan. The fly posterior midgut, which is functionally equivalent to the mammalian small intestine, is maintained by a dedicated population of intestinal stem cells (ISCs) (Figure 1B). The ISC activity is tightly regulated and coordinated to facilitate homeostatic as well as stress-induced epithelial renewal throughout an organism's lifetime (55–57). Interestingly, the posterior midguts of *ecd^1^* mutant adult flies kept at restrictive temperature for 15 days were markedly thinner compared to controls (Figure 1B–E) containing fewer large absorptive enterocytes (ECs). In contrast, the number of apoptotic progenitors (ISCs and enteroblasts (EBs)) and enteroendocrine cells (EEs) was significantly increased as determined by immunostaining against the activated *Drosophila* Cleaved Death caspase 1 (Dcp-1) (Figure 1F–H). Moreover, RNAi silencing of the *ecd* transcript specifically in the ISCs and EBs of adult flies for 20 days, using the *escargot-Gal4, UAS-GFP, tub-Gal80^TS^* system (hereafter abbreviated as *esg^TS^>*) (58) resulted in a decrease of Esg-positive progenitor cells, which markedly contrasted with their expansion in the age-matched control guts (Figure 1I–K). These results highlight a vital role of Ecd in the maintenance of tissue function and homeostasis during adulthood. Moreover, the changes in the gut morphology inflicted by *ecd* loss and the fact that intestinal cells demand Ecd function for their survival indicate that impaired renewal of the intestinal epithelium might be among the contributing factors to the lifespan shortening.

### *Ecd* deficiency causes decrease in Prp8 protein levels

**Figure 2.**
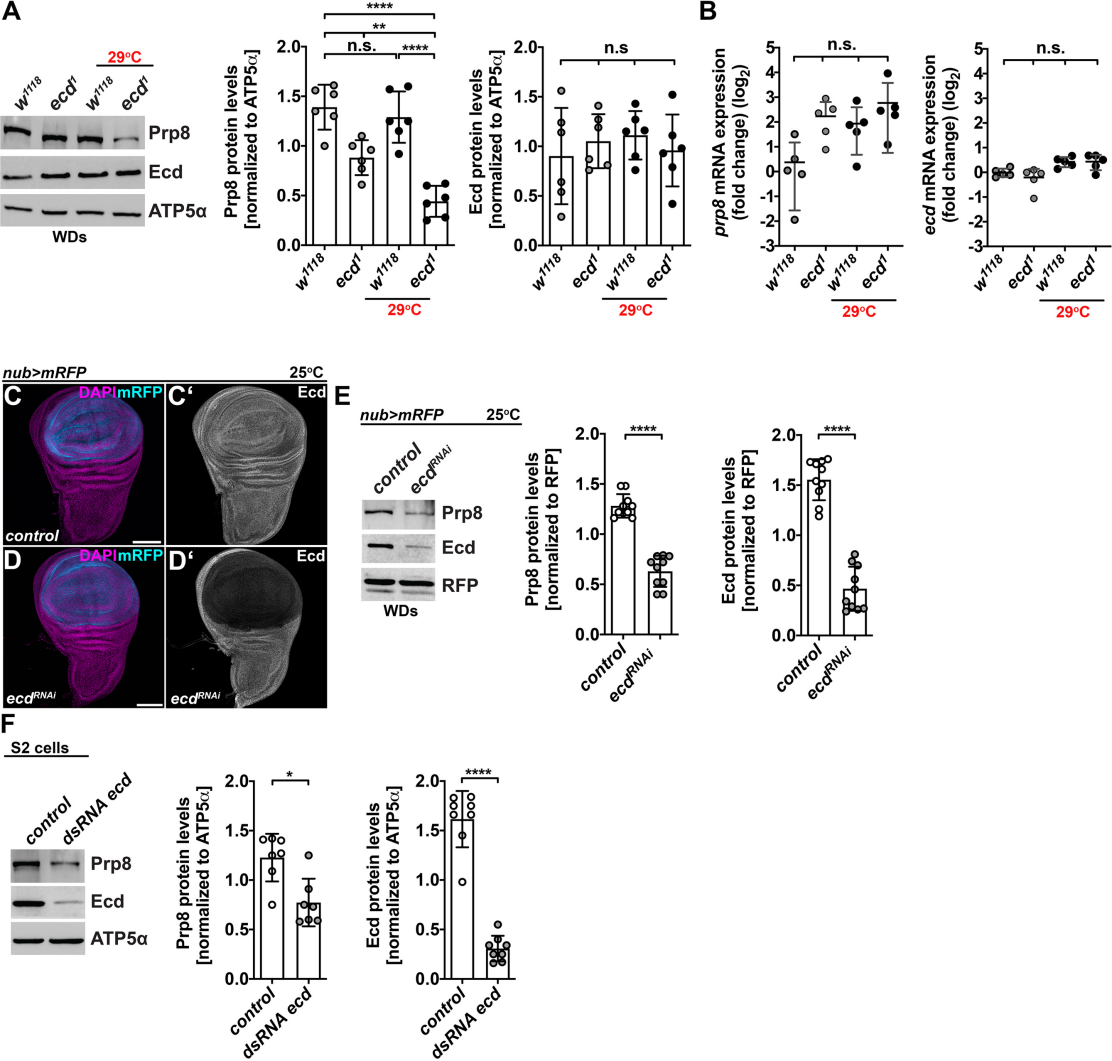
*Ecd* deficiency leads to Prp8 instability. (**A**) Representative western blot and quantifications show that Prp8 but not Ecd protein levels are reduced in wing imaginal discs (WDs) dissected from homozygous mutant *ecd^1^* larvae upshifted to 29°C for 24 h relative to controls. Note a noticeable decrease in Prp8 protein already observed at permissive temperature (18°C). ATP5α was used as a loading control. Data represent means ± SD, *n* = 6. Statistical significance was determined by two-way ANOVA with Tukey's multiple comparisons test, **P < 0.01, ****P < 0.0001, n.s. = non-significant. (**B**) *Prp8* and *ecd* mRNA levels did not differ significantly between WDs dissected from control and *ecd^1^* homozygous mutant larvae grown at permissive or restrictive temperature. Levels of *rp49* transcript were used for normalization. RT-qPCR data are means ± SD, *n* = 5. Statistical significance was determined by two-way ANOVA with Tukey's multiple comparisons test, n.s. = non-significant. (**C**, **D**) Immunostaining with an Ecd-specific antibody revealed marked reduction of Ecd protein in the pouch region of WDs following overexpression of an *UAS-ecd^RNAi^* transgene (D, D’) using the *nubbin-Gal4*, *UAS-myr-mRFP* driver (*nub>mRFP*) relative to hinge region and control WD (C, C’). Micrographs show projections of multiple confocal sections of WDs dissected from third instar larvae 7 days AEL. Nuclei were counterstained with DAPI. Scale bars: 100 μm. (**E**) Representative western blot and quantifications show a strong reduction of Prp8 and Ecd protein levels following RNAi-mediated knockdown of Ecd in the wing pouch using *nub>mRFP* driver. Red fluorescent protein (RFP) was used as a loading control. Data represent means ± SD, *n* = 10. Statistical significance was determined by paired two-tailed Student's *t*-test, ****P < 0.0001. (**F**) Prp8 protein levels decreased in S2 cells treated with *ecd* dsRNA compared to those transfected with control *lacZ* dsRNA for 72 h prior to lysis. ATP5α was used as a loading control. Data represent means ± SD, *n* = 7–8. Statistical significance was determined by paired two-tailed Student's *t*-test, *P < 0.05, ****P < 0.0001. See also Supplementary Figure S2.

To investigate the molecular consequences of Ecd loss, we concentrated on its interaction with the core U5 splicing factor Prp8. Intriguingly, western blot analysis revealed a marked downregulation of Prp8 protein in homozygous *ecd^1^* mutant adult flies kept at a restrictive temperature for 10 days (Supplementary Figure S2A). Similarly, Prp8 protein levels were decreased in wing imaginal discs (WDs) and eye-antennal imaginal discs (EADs) dissected from *ecd^1^* mutant larvae, which were exposed to 29°C for 24 h, relative to controls and levels detected at a permissive temperature (Figure 2A and Supplementary Figure S2B). In contrast, neither the P656S mutation nor the temperature shift significantly altered the levels of Ecd protein (Figure 2A and Supplementary Figure S2A, S2B) or the expression of *ecd* and *prp8* mRNAs, as determined by the quantitative RT-PCR (Figure 2B). Less Prp8 protein was also observed in WDs, in which Ecd was downregulated specifically in the pouch region via transgenic RNAi (*ecd^RNAi^*) under the control of the *nubbin-Gal4*, *UAS-myr-mRFP* driver (hereafter referred to as *nub>mRFP*) (Figure 2C–E) or in *Drosophila* S2 cells treated with a double-stranded RNA targeting the *ecd* transcript (*dsRNA ecd*) (Figure 2F). Interestingly, inhibition of the autophagy-lysosomal degradation pathway with Chloroquine (CQ) or Bafilomycin A1 (BafA1) restored the Prp8 protein levels in *ecd* deficient S2 cells to amounts detected in controls (Supplementary Figure S2C, S2D). These results demonstrate that the loss of Ecd causes Prp8 protein instability and suggest that Ecd protects Prp8 from degradation.

### Mutant Ecd proteins preserve binding to R2TP complex

**Figure 3.**
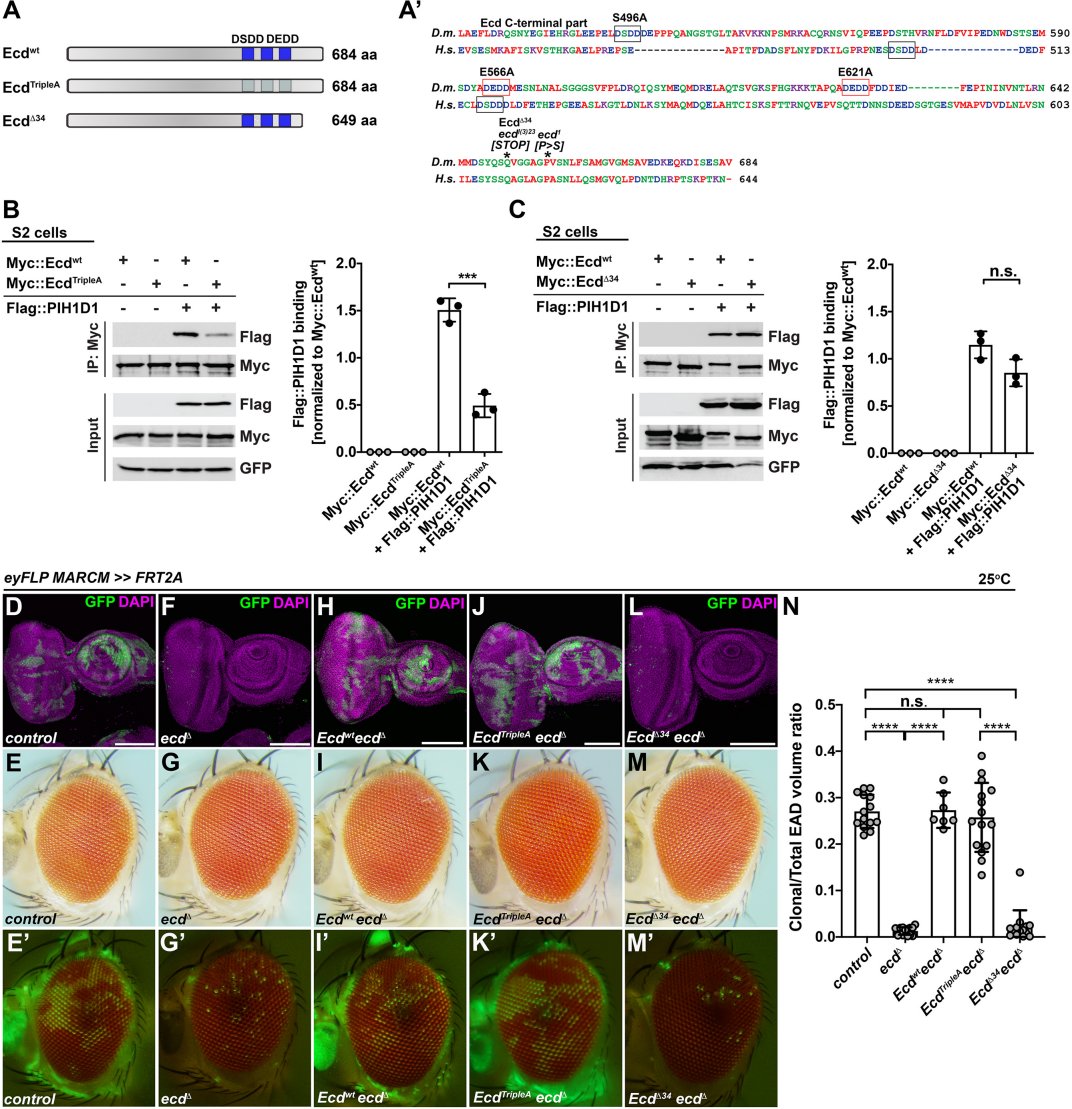
Ecd^Δ34^ mutant protein binds R2TP complex via PIH1D1 but is non-functional *in vivo*. (**A**) Schematic representation of wild-type and mutant Ecd proteins and *ecd* alleles used in this study. The blue boxes indicate the position of wild-type (dark blue) or mutated (light blue) DSDD/DEDD motifs. Alignment of the C-terminal part of the *Drosophila melanogaster* (*D.m*., Q9W032) Ecd and human (*H.s.*, O95905) ECD proteins was generated using Clustal W (A’). Asterisks indicate the positions of the premature stop codon in *ecd^l(^^3^^)23^* allele and the conserved proline 656, which substitution to serine generates conditional *ecd^1^* allele. Black and red rectangles outline the DSDD and DEDD motifs, respectively (A’). (**B**, **C**) Representative western blots and quantifications show that Myc::Ecd^TripleA^ (B) but not Myc::Ecd^Δ34^ (C) co-precipitates significantly less Flag::PIH1D1 protein from *Drosophila* S2 cell lysates relative to Myc::Ecd^wt^ (B, C). Myc-tagged proteins served as baits. GFP was used as a transfection and loading control. Data represent means ± SD, n = 3. Unpaired two-tailed Student's *t*-test was used to determine the significance, ***P < 0.001, n.s. = non-significant. (**D–M**) Representative confocal micrographs of mosaic third instar EADs and brightfield and fluorescent images of adult eyes, where homozygous GFP-labelled clones of the indicated genotypes were generated using the eyFLP MARCM technique. In contrast to abundant, sizable control clones (D, E), *ecd^Δ^* homozygous mutant clones are very rare, presented as individual GFP-positive cells within the differentiated part of the eye primordium (F) and adult retina (G). Overexpression of Ecd^wt^ (H, I) and Ecd^TripleA^ (J, K) but not Ecd^Δ34^ mutant protein (L, M) is sufficient to restore clonal number and size to control levels (D, E). Confocal micrographs are projections of multiple sections, showing EADs 7 days AEL. Nuclei were counterstained with DAPI. Scale bars: 100 μm (D, F, H, J, L). (**N**) Quantification of clonal to total EAD volume ratios from confocal micrographs of mosaic EADs of the indicated genotypes. Data represent means ± SD, *n* = 7–16. Ordinary one-way ANOVA with Tukey's multiple comparisons test was used to determine significance, ****P < 0.0001, n.s. = non-significant. See also Supplementary Figure S3.

Biochemical and cell culture experiments showed that human ECD interacts with the core subunits of the R2TP chaperone complex (25,26), which has been implicated in the assembly of the functional U5 snRNPs (19,23). We therefore hypothesized that Ecd might promote the maturation of U5 snRNPs by acting as a molecular adapter between the U5 snRNP and the R2TP complex. In the absence of Ecd on the other hand, the U5 snRNP assembly would be compromised, leaving Prp8 exposed and vulnerable to degradation. Importantly, co-IP experiments from *Drosophila* S2 cells expressing the tagged proteins revealed binding of Ecd to the known R2TP subunits, PIH1D1 and Pontin (RUVBL1) (Figure 3B, 3C and Supplementary Figure S3A, S3B), demonstrating that the reported interactions between Ecd and R2TP complex in mammalian cells (19,25,26) can be recapitulated in *Drosophila*. To assess the importance of Ecd engagement with R2TP, we decided to perturb the interaction by mutating the D[S/T]DD motifs, which facilitate the recruitment of Ecd to the PIH-N domain of PIH1D1 likely in a phospho-dependent manner (26). The alignment of fly and human proteins revealed one potential interacting D[S/T]DD motif in *Drosophila* Ecd. In addition, we identified two DEDD amino acid stretches, which could mimic the phosphorylation of the D[S/T]DD motif (Figure 3A). To test the functional significance of these motifs, we exchanged the phosphorylatable Ser (S496) and phosphomimetic Glu (E556, E621) residues with Ala generating an Ecd^TripleA^ mutant version (Figure 3A). While the presence of three Ala residues compromised binding to PIH1D1, the amount of Pontin pulled down by Ecd^TripleA^ was comparable to the precipitation efficiency of the wild-type Ecd (Ecd^wt^) (Figure 3B and Supplementary Figure S3A). To determine the impact of the point mutations *in vivo*, we took advantage of the Mosaic analysis with a repressible marker (MARCM) technique (38) to overexpress the Ecd^TripleA^ mutant protein in the GFP-labeled *ecd* homozygous mutant cells induced in EADs by *eyeles*s-FLP-mediated recombination. To this end, we used the CRISPR-Cas9 genome editing tool to generate an *ecd* loss-of-function allele (*ecd*^Δ^), which lacks almost the entire open reading frame except the first twenty-three amino acids (Supplementary Figure S1A). In contrast to control, only a few isolated GFP-positive homozygous *ecd*^Δ^ cells were present in the third instar EADs and adult eyes (Figure 3D–G, 3N and Supplementary Figure S1B, S1F), which is consistent with the lethality of cells homozygous for a non-conditional *ecd^l(^^3^^)23^* (22 and Supplementary Figure S1G) and a conditional *ecd^1^* allele (Supplementary Figure S1C, S1E). Importantly, overexpression of Ecd^TripleA^ rescued the viability and growth of *ecd* deficient cells resulting in clones that were comparable in size and number to control and *ecd*^Δ^ clones overexpressing Ecd^wt^ (Figure 3H–K, 3N). Interestingly, overexpression of Ecd^Δ34^ protein variant that mimics a truncated product generated from the lethal *ecd^l(^^3^^)23^* allele (Figure 3A andSupplementary Figure S1A) failed to rescue the viability of the homozygous *ecd*^Δ^ mutant EAD cells (Figure 3L–N) while it retained the capacity to bind the R2TP components in S2 cells (Figure 3C and Supplementary Figure S3B). Of note, the overexpression of Ecd^TripleA^ or Ecd^Δ34^ in wild-type EAD cells had no visible phenotypical consequences with mosaic EADs and adult eyes being indiscernible from controls (Supplementary Figure S3C–K).

These results demonstrate that the interactions between Ecd and the two components of the R2TP chaperon complex, Pontin and PIH1D1, are evolutionarily conserved although the binding to PIH1D1 might require other motifs besides D[S/T/E]DD. Importantly, the genetic *in vivo* experiments argue against a primary function of Ecd as a linker between the assembling U5 snRNPs and R2TP while highlighting the indispensable role for the C-terminal part of the Ecd protein for cell viability.

### Ecd interacts with proteins of the heptameric Sm ring

**Figure 4.**
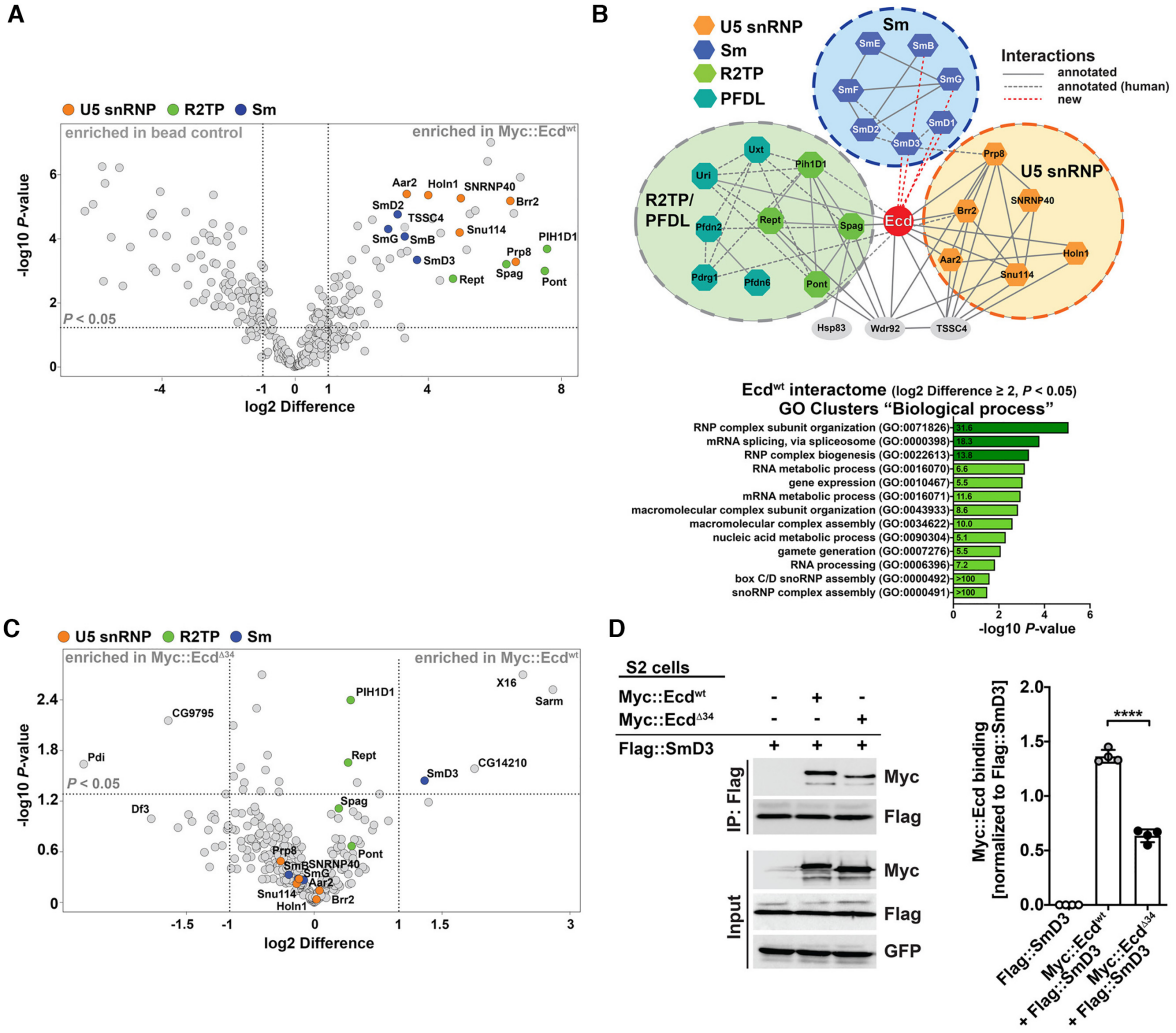
The heptameric Sm ring protein SmD3 interacts with Ecd. (**A**) The volcano plot depicts Ecd-specific interactome identified by LC-MS/MS analysis based on pull-down of Myc::Ecd^wt^ protein from *Drosophila* S2 cell lysate with an anti-Myc-specific antibody. 61 proteins enriched in Myc::Ecd^wt^ IP samples relative to control (S2 cells not expressing Myc::Ecd^wt^) (|log_2_ difference| ≥ 1 and FDR < 0.05) are located within the outlined area. Specific interaction partners including Sm proteins (blue), U5 snRNP proteins (orange), and R2TP components (green) are highlighted. (**B**) Visualization of a simplified Ecd interaction network comprising Sm (blue), U5 snRNP (orange) and R2TP complex (green) proteins using Cytoscape. Annotated interactions are shown with full or dashed gray lines, new interactions with red dashed lines. The bar graph depicts functional GO clusters enriched (fold enrichment ≥ 5, FDR < 0.05, noted within the bars) among the Myc::Ecd^wt^ interacting proteins (|log_2_ Difference| ≥ 2 and FDR < 0.05). GO terms linked to RNP assembly and RNA splicing are highlighted in dark green. (**C**) The volcano plot depicts results of comparative LC–MS/MS analysis showing differences in proteins pulled down with Myc::Ecd^wt^ and Myc::Ecd^Δ34^ from *Drosophila* S2 cell lysates. Sm proteins (blue), U5 snRNP proteins (orange), and R2TP components (green) are highlighted. Note that the binding of SmD3 to Ecd^Δ34^ protein is markedly reduced. (**D**) Representative western blot and quantifications of independent IP assays in *Drosophila* S2 cells confirm decreased binding of Flag::SmD3 to Myc::Ecd^Δ34^ compared to Myc::Ecd^wt^ protein. Proteins were pulled down by anti-Flag magnetic beads. Data represent means ± SD, *n* = 4. Unpaired two-tailed Student's *t*-test was used to determine significance, ****P < 0.0001. See also Supplementary Figure S4 and Supplementary Dataset S1.

The fact that the truncated Ecd^Δ34^ variant was able to interact with the R2TP complex components but failed to functionally substitute for the wild-type protein *in vivo*, prompted us to take an unbiased approach to investigate the protein interactome of Ecd^wt^ and compare it to that of Ecd^Δ34^. To this end, we expressed the Myc-tagged Ecd^wt^ and Ecd^Δ34^ in S2 cells and performed IP experiments followed by liquid chromatography–tandem mass spectrometry (LC–MS/MS) analysis. The Ecd^wt^ interactome consisted of 61 proteins that were significantly enriched (|log_2_ Difference| ≥ 1 and *P*-value < 0.05) compared to the mock samples (Figure 4A and Supplementary Dataset S1). The Gene Ontology (GO) enrichment and clustering analyses revealed an overrepresentation of terms associated with biological functions related to RNP assembly and organization, RNA splicing, nucleic acid metabolism and gene expression (Figure 4B). In addition to already described Ecd binding partners, such as core constituents of the U5 snRNP and the R2TP/PFDL co-chaperone complex (Figure 4A, 4B and Supplementary Dataset S1), we identified the proteins of the heptameric Sm ring, SmD3, SmB, SmD1 and SmG, as plausible novel interacting partners of Ecd (Figure 4A, 4B). The direct binding of Ecd to SmD3 but not SmB, SmG or SmF was further validated by independent co-IP experiments in S2 cells and a pull-down assay using affinity purified recombinant His-tagged Ecd and SmD3 proteins expressed in bacteria (Figure 4D and Supplementary Figure S4A, S4B). Importantly, the comparative MS analysis and follow-up co-IP experiments showed that SmD3 binding to a truncated Ecd^Δ34^ protein was markedly reduced relative to Ecd^wt^, (Figure 4C, 4D and Supplementary Dataset S1) while the interactions of Ecd^wt^ and Ecd^Δ34^ with the R2TP complex components (PIH1D1, Pontin) and Prp8 were not significantly different (Figures 4C, 3C, Supplementary Figure S3B and Supplementary Dataset S1).

Taken together, our proteomic approach identified known as well as novel interaction partners of Ecd including the core components of the U5 snRNP complex, Prp8 and Sm ring proteins, respectively. We further demonstrated that the binding of Ecd to SmD3 is direct and that the intact C-terminus of the Ecd protein is required for productive binding to the assembling U5 snRNP.

### Ecd malfunction interferes with U5 snRNP biogenesis

**Figure 5.**
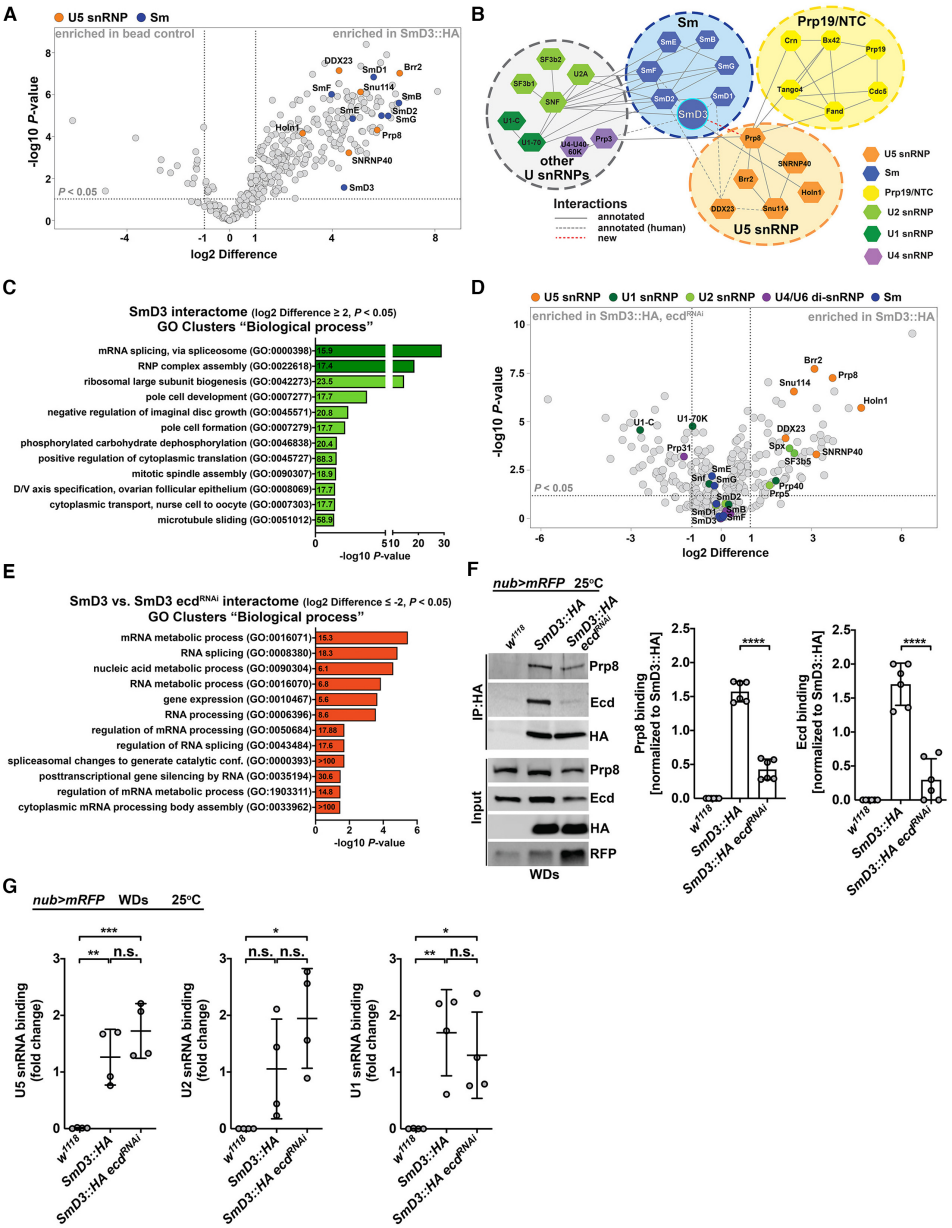
*Ecd* deficiency hinders U5 snRNP biogenesis. (**A**) The volcano plot depicts SmD3-specific interactome identified by LC–MS/MS analysis based on a pull-down of SmD3::HA protein from *nub>mRFP, SmD3::HA* wing imaginal disc cell lysate. 252 proteins enriched in SmD3::HA IP samples relative to control (*nub>mRFP*) (|log2 Difference| ≥ 1 and FDR < 0.05) are located within the outlined area. Specific interaction partners including Sm proteins (blue), U5 snRNP proteins (orange) are highlighted. (**B**) Visualization of a simplified SmD3 protein interactome using Cytoscape highlights its central position within the Sm ring and the spliceosome. Annotated interactions are shown with full or dashed gray lines, new interactions with green dashed lines. (**C**) The bar graph shows functional GO clusters enriched (fold enrichment ≥ 15, FDR < 0.05, depicted within the bars) among the SmD3::HA interacting proteins (|log_2_ difference| ≥ 2 and FDR < 0.05). GO terms linked to RNP assembly and RNA splicing are highlighted in dark green. (**D**) The volcano plot visualizes results of a comparative LC–MS/MS analysis showing differences in protein binding to SmD3::HA precipitated from control (*nub>mRFP, SmD3::HA*) wing discs lysates and those where *ecd* was knocked down (*nub>mRFP, SmD3::HA, ecd^RNAi^*). RNAi-mediated silencing of *ecd* transcript reduces interaction of SmD3 with numerous core components of the U5 snRNP (orange) but not with other Sm proteins comprising the Sm ring (blue). The interactions of SmD3 with U1 (dark green), U2 (light green) and U4/U6 snRNP-specific proteins were less affected compared to the components of U5 snRNP. (**E**) The bar graph shows functional GO clusters enriched (fold enrichment ≥ 5, FDR adjusted < 0.05, depicted within the bars) among the SmD3::HA interacting proteins that are significantly affected (|log_2_ difference| ≥ 2 and FDR < 0.05) by *ecd* RNAi knockdown. (**F**) Representative western blot and quantifications of independent IP experiments corroborate reduced binding of endogenous Prp8 to SmD3::HA in lysates prepared from *ecd* depleted (*nub>mRFP, SmD3::HA*, *ecd^RNAi^*) wing imaginal discs compared to control *nub>mRFP, SmD3::HA* samples. Proteins were pulled down with help of the anti-HA antibody-coupled to magnetic beads. Imaginal discs expressing RFP only (*nub>mRFP*) served to control for unspecific binding. Data represent means ± SD, *n* = 6. Unpaired two-tailed Student's *t*-test was used to determine significance, ****P < 0.0001. (**G**) RIP assays from wing imaginal disc samples using the anti-HA antibody-coated magnetic beads reveal that transgenic SmD3::HA protein precipitates endogenous *U5*, *U2* and *U1 snRNAs* in the presence (*nub>mRFP, SmD3::HA*) as well as absence of *ecd* (*nub>mRFP, SmD3::HA, ecd^RNAi^*). Imaginal discs expressing RFP only (*nub>mRFP*) served to control for unspecific binding. *U snRNA* levels were determined by RT-qPCR. Data were normalized to the respective input *U snRNA* levels. Data represent means ± SD, *n* = 4. Ordinary one-way ANOVA with Tukey's multiple comparisons test was used to determine significance, *P < 0.05, **P < 0.01, ***P < 0.001, n.s. = non-significant. See also Supplementary Figure S5 and Supplementary Dataset S2.

The ability of Ecd to precipitate Prp8 but also SmD3 and the fact that Prp8 levels decrease in its absence further strengthen the notion that Ecd plays a role in U5 snRNP biogenesis. To determine whether Ecd dysfunction impacts U5 snRNP composition, we expressed the HA-tagged SmD3 protein (SmD3::HA) in the wing imaginal cells under the control of the *nub>mRFP* driver that were either wild-type or deficient for Ecd due to RNAi-mediated silencing. We immunoprecipitated SmD3 interacting proteins from lysates prepared from the dissected WDs with a HA-specific antibody and analyzed them by LC–MS/MS. Consistent with its vital role in building the heptameric Sm ring around the Sm site of *U1*, *U2*, *U4* and *U5 snRNAs*, SmD3 pulled down all the other six subunits (SmB, SmD1, SmD2, SmE, SmF and SmG) (Figure 5A, 5B and Supplementary Dataset S2). In addition, the SmD3 interactome contained numerous U snRNP specific proteins, including U5-type splicing proteins, which was also reflected in the enrichment of GO categories associated with RNP complex assembly and pre-mRNA splicing (Figure 5A-C and Supplementary Dataset S2). Strikingly, the binding of SmD3 to the core components of the U5 snRNP, including Prp8, I(3)72Ab (Brr2), Snu114, Holn1, CG3436 (SNRNP40) and CG10333 (DDX23) was strongly reduced when Ecd function was compromised (Figure 5D, 5F, Supplementary Figure S5A and Supplementary Dataset S2). In agreement, the GO categories comprising of these proteins were particularly affected and significantly different between control and *ecd* deficient samples (Figure 5E and Supplementary Dataset S2). In contrast, Ecd loss did not compromise interactions of SmD3 with other Sm ring subunits (Figure 5D and Supplementaty Dataset S2) and *U snRNAs*, including *U5*, *U1* or *U2 snRNA*, as determined by RNA immunoprecipitation (RIP) assays (Figure 5G), suggesting that Prp8 incorporation to the U5 snRNP follows the Sm rings assembly around the *U5 snRNA*.

**Figure 6.**
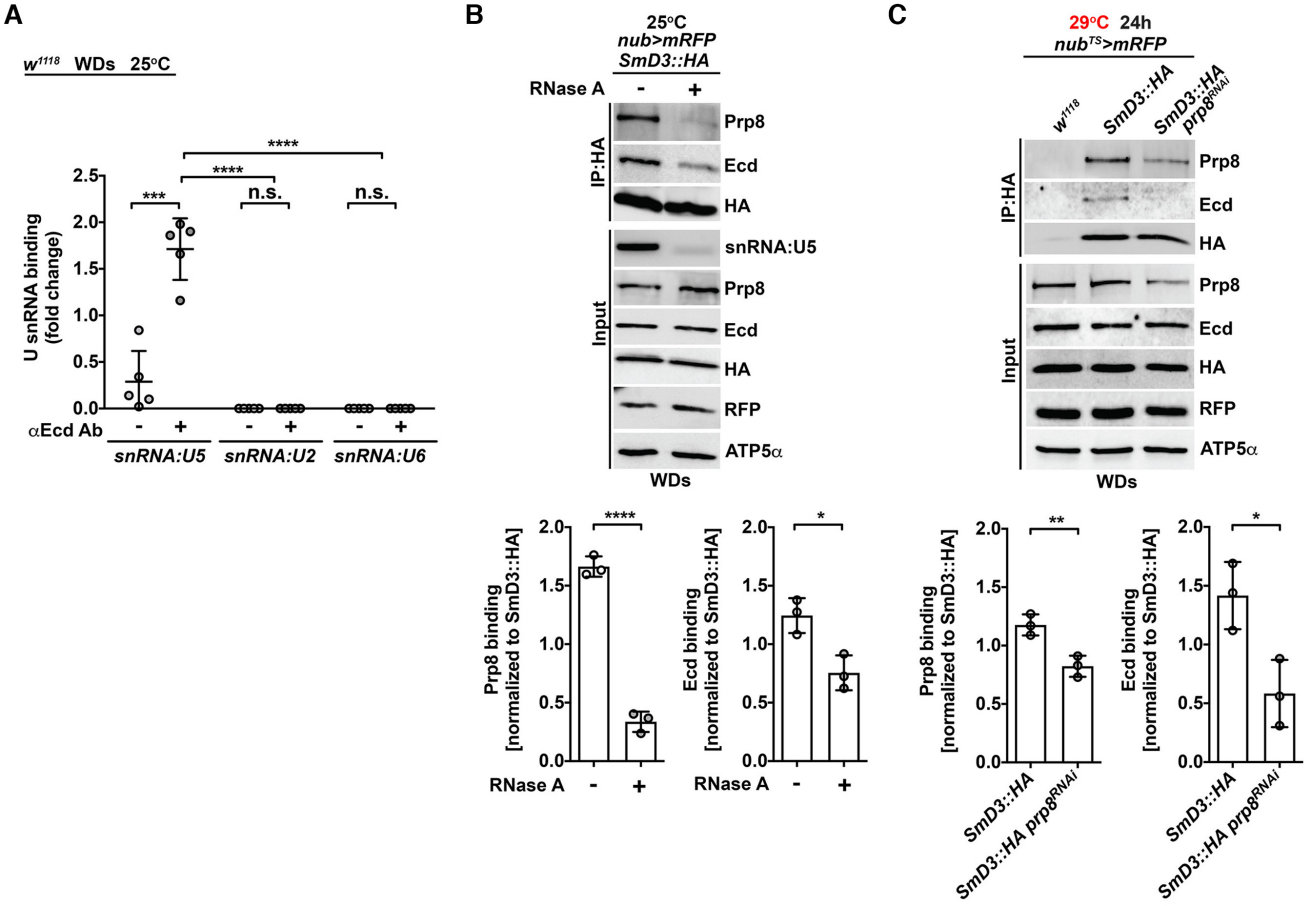
Ecd binding to SmD3 in the U5 snRNP requires Prp8 and the *U5 snRNA*. (**A**) RIP assays from control (*w^1118^*) wing imaginal discs (WDs) with a Ecd-specific antibody show selective binding of endogenous Ecd to *U5* but not *U2* and *U6 snRNAs*. The amount of precipitated *U snRNAs* was quantified by RT-qPCR and normalized to the respective input *U snRNA* levels. Data represent means ± SD, *n* = 5. Statistical significance was determined using two-way ANOVA with Tukey's multiple comparisons test, ***P < 0.001, ****P < 0.0001, n.s. = non-significant. (**B**) Representative western blot and quantification of independent IP experiments show reduced binding of endogenous Prp8 and Ecd to transgenic SmD3::HA in RNAse A treated (+) lysates prepared from *nub>mRFP, SmD3::HA* wing imaginal discs compared to untreated (–) samples. Proteins were precipitated with the anti-HA antibody-coated magnetic beads. Semi-quantitative PCR of *snRNA:U5* shows RNase A treatment efficacy. Data represent means ± SD, *n* = 3. Unpaired two-tailed Student's *t*-test was used to determine significance, *P < 0.05, ****P < 0.0001. (**C**) Representative western blot and quantification of independent IP experiments using the HA antibody-coated magnetic beads show reduced binding of endogenous Ecd to a transgenic SmD3::HA protein in *prp8* depleted (*nub^TS^>mRFP, SmD3::HA prp8^RNAi^*) wing imaginal disc lysates compared to *nub^TS^>mRFP, SmD3::HA* samples. Imaginal discs expressing RFP only (*nub^TS^>mRFP*) served to control for unspecific binding. The transgene expression was induced for 24 h by temperature inactivation of Gal4 repressor Gal80. Data represent means ± SD, *n* = 3. Statistical significance was determined using unpaired two-tailed Student's *t*-test, *P < 0.05, **P < 0.01. See also Supplementary Figure S6

To further delineate why the disruption of Ecd-SmD3 interaction is particularly detrimental to U5 snRNP biogenesis rather than affecting assembly of all Sm-ring-containing snRNPs, we tested for interdependency of specific protein-protein interactions as well as the role of *U snRNAs*. Intriguingly, the RIP assay showed exclusive precipitation of the *U5 snRNA* but no other tested *U snRNAs* with Ecd (Figure 6A). Moreover, the RNA presence supported efficient binding of Ecd to SmD3 as the interaction weakened when S2 cell lysates were pretreated with RNAse A (Figure 6B). While the Prp8 association with the core U5 snRNP requires Ecd, the fact that significantly less Ecd precipitated with SmD3 when imaginal cells were temporarily depleted of Prp8 using the *nubbin-Gal4, UAS-myr-mRFP, tub-Gal80^TS^*system (hereafter referred to as *nub^TS^>mRFP*) (Figure 6C) suggests that Ecd and Prp8 are mutually dependent on each other for effective binding to SmD3. Enhanced interaction between Ecd and SmD3 in imaginal cells overexpressing Prp8 relative to control further supports the cooperative mode of U5 snRNP assembly (Supplementary Figure S6A).

Taken together, our results demonstrate the specific requirement for Ecd during later steps of U5 snRNP biogenesis while the formation of an intact Sm ring on the *U5 snRNA* is Ecd-independent. The efficient loading of Prp8 onto the assembling U5 snRNP particles relies on Ecd and its interaction with SmD3 and *U5 snRNA* while *ecd* loss aborts the biogenesis process resulting in Prp8 instability.

### *Ecd* deficiency causes transcriptome-wide changes in gene expression and splicing

**Figure 7.**
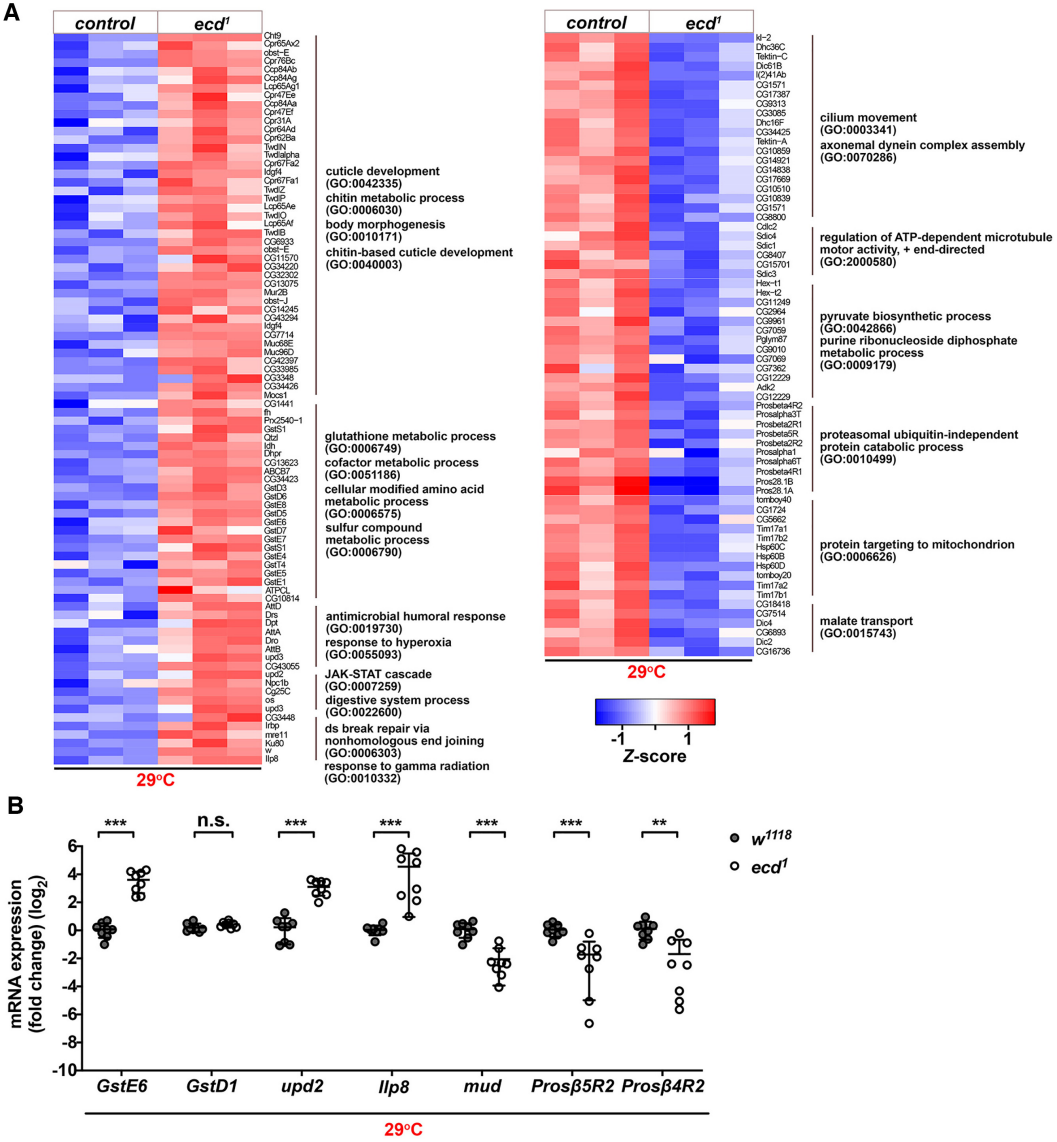
*Ecd* deficiency leads to transcriptome-wide changes in gene expression. (**A**) Heatmaps visualize relative expression levels of genes up and downregulated in homozygous mutant *ecd^1^* larvae relative to control (*w^1118^*) upshifted to 29°C, which are clustered according to enriched GO terms (fold enrichment ≥ 3, *P* < 0.05) between individual samples. Data are log_2_(*x* + 1) transformed normalized counts. The color scale represents the *Z*-scores for each gene and each column represents one biological replicate of the indicated genotype. (**B**) Independent RT-qPCR confirms upregulation of stress-related genes (*GstE6*,*upd2, Ilp8*) in homozygous mutant *ecd^1^* larvae relative to control (*w^1118^*) upshifted to 29°C while expression of proteasome subunits (*Prosß5R2*, *Prosß4R2*) and a regulator of microtubule organization (*mud*) was downregulated. Levels of *rp49* transcripts were used for normalization. Data represent means ± SD, *n* = 8. Unpaired two-tailed Student's *t*-test with Welch's correction was used to determine significance, **P < 0.01, ***P < 0.001, n.s. = non-significant. See also Supplementary Figure S7 and Supplementary Dataset S3.

The marked changes in U5 snRNP composition as a consequence of *ecd* loss imply that cells might suffer from insufficient amount of functional snRNP particles. Given the mutual coupling between splicing and transcription, such spliceosome deprivation would likely manifest in broad changes in gene expression and pre-mRNA splicing pattern. Indeed, the transcriptome profiling of *ecd^1^* homozygous larvae at restrictive temperature revealed misexpression of 2952 genes among which 771 genes were upregulated while 2181 were downregulated relative to control using |log_2_ FC| ≥ 1 and adjusted *P*-value < 0.05 as a cut off (Figure 7A and Supplementary Dataset S3). The follow-up GO clustering analysis highlighted that genes associated with responses to various stress stimuli, DNA repair, digestive system processes, and glutathione, chitin and amino acid metabolism were markedly enriched among transcripts upregulated in *ecd^1^* mutant larvae. In contrast, downregulated genes were associated with functions related to ‘proteasomal catabolic processes’, ‘pyruvate, purine and malate metabolism’, ‘mitochondria transport’ and ‘microtubule motor protein assembly and function’ (Figure 7A and Supplementary Figure S7A and Supplementary Dataset S3). These data indicate that *ecd* deficiency causes energy imbalance, loss of metabolic and redox homeostasis and activation of global stress response. Importantly, the differential expression of several candidates was validated by RT-qPCR on independent samples. Compared to control *GstE6, upd2, Ilp8* were increased in *ecd^1^* samples while the levels of *mud*, *Prosβ5R2* and *Prosβ4R2* were significantly downregulated at 29°C but not at permissive temperature (Figure 7B and Supplementary Figure S7B). Although expression of the major *U snRNAs* showed rising trend in imaginal tissues of *ecd^1^* at the restrictive temperature none of these changes appeared significant when compared to the respective control samples (*w^1118^* at 29°C) (Supplementary Figure S7C).

**Figure 8.**
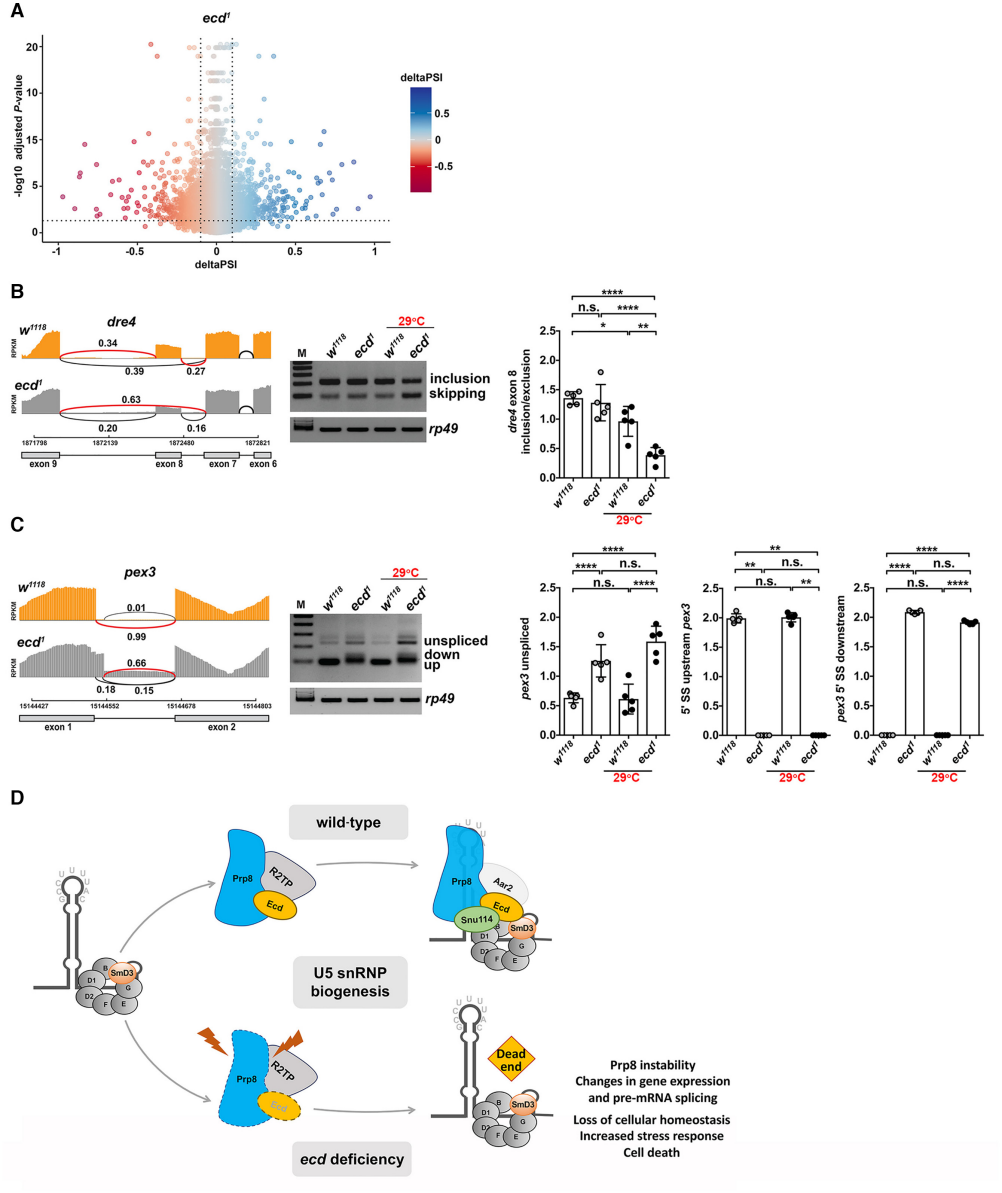
*Ecd* deficiency causes global alterations in splicing pattern. (**A**) The volcano plot shows changes in splicing pattern in *ecd^1^* homozygous mutant third instar larvae upshifted to 29°C versus control (*w^1118^*) identified by *LeafCutter*. Every dot represents a splicing event. Dotted lines demarcate significantly changed events with delta percent spliced in deltaPSI ≥ 0.1 and adjusted *P*-value ≤ 0.05. (**B**, **C**) Sashimi-plots of selected candidate genes *dre4* (B) and *pex3* (C) from mRNA-seq data show alterations of splicing patterns in *ecd^1^* homozygous mutant larvae compared to control (*w^1118^*). The representative images of semiQ-PCR gels and quantifications confirm missplicing events in *dre4* (B) and *pex3* (C) in *ecd^1^* homozygous mutant wing discs relative to control dissected from third instar larvae that were kept at 18°C or upshifted to non-permissive temperature (29°C). Note that alteration to *pex3* pre-mRNA splicing is already apparent in *ecd^1^* samples at 18°C. Levels of *rp49* transcripts were used for normalization. Data represent means ± SD, *n* = 5. Two-way ANOVA with Tukey's multiple comparison test was used to determine significance, *P < 0.05, **P < 0.01, ****P < 0.0001, n.s. = non-significant. (**D**) Proposed model for the role of Ecd in the cytoplasmic part of U5 snRNP biogenesis. U5 snRNP maturation is a step-wise process that starts in cytoplasm with SMN-guided assembly of the heptameric Sm protein ring around the conserved Sm binding site on the *U5 snRNA*. The generated core U5 snRNP particle then serves as a platform for association of further U5 snRNP-specific proteins. Ecd functions as a chaperon that, through interaction with SmD3, brings Prp8. The effective U5 snRNP biogenesis relies on binding cooperativity of Ecd and Prp8 and interactions with *U5 snRNA*. In the absence of Ecd, the core U5 snRNP particle forms properly, but Prp8 protein remains unshielded and destabilized, which interferes with U5 snRNP maturation, ultimately leading to spliceosome scarcity, transcriptome-wide splicing errors and cell death. See also Supplementary Figure S7 and Supplementary Dataset S4.

The marked shift in gene expression was accompanied with changes in splicing patterns of numerous genes as determined by the *LeafCutter* algorithm (51) (Figure 8A and Supplementary Dataset S4). We identified alterations in all known types of splicing events, including exon skipping (ES), as well as alternative 5′ and 3′ splice site selection (Supplementary Figure S7D and Supplementary Dataset S4), suggesting systemic dysregulation of pre-mRNA splicing. Interestingly, the misspliced genes did not show enrichment for specific functional categories. An independent RT-PCR on selected candidates confirmed missplicing events upon *ecd* loss including exon skipping in *dre4*, encoding a chromatin regulator of the Facilitates Chromatin Transcription (FACT) complex (Figure 8B), as well as intron retention and changes in splice site choice in *peroxin 3* (*pex3*), which is essential for peroxisome biogenesis (Figure 8C). Taken together, these results demonstrate that Ecd dysfunction causes genome-wide changes in gene expression and splicing aberrations consistent with a more general role of Ecd in governing U5 snRNP biogenesis (Figure 8D).

## DISCUSSION

The dynamic regulation of gene expression during the development and adult life of multicellular organisms requires highly effective and accurate splicing machinery. To produce functional spliceosomes, individual snRNPs undergo a stepwise assembly process that is tightly controlled in time and space. In this study, we combined functional genetics, proteomic and genomic approaches to define an evolutionarily conserved protein Ecd as a crucial mediator of the U5 snRNPs assembly that guards the delivery of the core splicing factor Prp8 to the Sm protein ring of forming snRNP particles (Figure 8D). In contrast, the *ecd* loss impairs Prp8 stability and U5 snRNP biogenesis, causing genome-wide alterations in gene expression and splicing.

Ecd was originally described through isolation of the conditional *ecd^1^* mutant allele in *Drosophila* (32,59). While the molting defects and developmental arrest of *ecd^1^*homozygous mutant larvae had been attributed to the systemic deficiency of the major steroid hormone ecdysone, functional experiments using genetic mosaics firmly established the cell-autonomous requirement of Ecd for cell survival (22,27 and this study). The cell-intrinsic role of Ecd has been further underscored by a number of recent studies in fly and mammalian cells reporting Ecd interactions with proteins of the splicing machinery, components of the U5 snRNP in particular, and the HSP90-R2TP/PFDL co-chaperone implicated in RNP assembly (19,22,24,26). Consistent with the proposed role of Ecd in pre-mRNA splicing, we now demonstrate that, like Prp8 (60), Ecd function remains vital beyond animal development to maintain tissue function and homeostasis during adulthood.

Our experiments in *Drosophila* S2 cells and larval tissues *in vivo* revealed the reduction of overall Prp8 protein levels as a common molecular feature of *ecd* loss, indicating that through binding to Ecd Prp8 might be stabilized and protected from undesired interactions. Interestingly, programmed downregulation of selected snRNP proteins to avoid their aggregation and proteotoxicity has been reported (19,61–63). For example, knockdown of PRMT5 complex subunits that facilitate the assembly of a toroidal Sm protein ring on the *U snRNAs* resulted in a transient backlog of Sm proteins and their subsequent lysosomal degradation in HeLa cells (61).

The function of Prp8 caretaker has been recently assigned to the Hsp90-R2TP/PFDL co-chaperone complex (19,25,26). As chemical inhibition of Hsp90 resulted in reduced Prp8 protein levels in mammalian cultured cells it has been proposed that R2TP binding stabilizes unassembled forms of Prp8 on their way towards newly forming U5 snRNP in the cytoplasm (19). Intriguingly, Ecd along with other proteins, including ZNHIT2 and TSSC4, were identified as putative interactors of the R2TP complex and Prp8 in HeLa cells (19,64) with ZNHIT2 being highlighted as the bridging factor between R2TP complex and Prp8 (23). Here we demonstrate that the interactions of Ecd with the R2TP complex are conserved in *Drosophila*. Like in mammalian cells, binding of *Drosophila* Ecd to PIH1D1 relies, at least in part, on the DSDD/DEDD motifs, while interaction with Pontin/RUVBL1 is not affected by the substitution of Ser/Glu with Ala. The genetic rescue experiments, however, provide functional evidence that the Ecd•R2TP assembly, is not essential *in vivo*. Although compromised in R2TP binding, the Ecd^TripleA^ mutant protein fully restored the viability and growth of *ecd* deficient imaginal disc cells. In contrast, the truncated Ecd^Δ34^ protein, which interactions with Pontin, PIH1D1, and Prp8 were not affected (22 and this study), failed to rescue. The fact that the Ecd^Δ34^ mutant protein retained the capacity to bind to and likely protect Prp8 from degradation strongly indicated that the Prp8 instability observed in *ecd* deficient cells (*ecd^1^*, *ecd^RNAi^*) represents a phenotypic hallmark but is not per se causative to the loss of cell viability. Intriguingly, our unbiased proteomic approach identified SmD3 as a novel binding partner of Ecd and demonstrated a pivotal role of the C-terminal part of the Ecd protein to mediate this interaction. Based on these findings, we, therefore, propose that the primary role of Ecd is to act as an adaptor between Prp8 sub-assemblies and SmD3 within the core U5 snRNP particle rather than between Prp8 and the R2TP complex. We further suggest that Ecd aids Prp8 stabilization, and their cooperative binding promotes efficient interaction with SmD3, while their selective affinity to *U5 snRNA* confers specificity to U5 snRNP assembly. Importantly, our data are not in conflict with the described role of R2TP/PRFD and its cofactors TSSC4 and ZNHIT2 in tethering and shielding Prp8 (19,23). Noteworthy, *Drosophila* TSSC4 ortholog, encoded by *Drosophila* CG6674, was present within the Ecd interactome. Yet, TSSC4 binding to Ecd^wt^ and the mutant Ecd^Δ34^ protein did not differ. It is tempting to speculate that Ecd and R2TP might cooperate to stabilize Prp8 in conditions or tissues, which demand higher production of U5 snRNPs. Such multivalent interactions between Prp8-associated proteins and the R2TP complex may provide robustness while securing a minimum stabilization of Prp8 in absence of either of the adaptors.

Consistent with its role in guiding Prp8 to the forming U5 snRNP, the interaction between SmD3 and Prp8 markedly weakened in cells lacking Ecd. In addition, the amount of all other U5 snRNP-specific proteins, including Brr2, Snu114, Holn1, snRNP40 and DDX23 precipitated by SmD3, were lower. These results support the notion that Ecd promotes U5 snRNP assembly while its loss leads to ‘dead-end’ U5 snRNP intermediates that cannot complete maturation. Given the multidomain structure of Prp8 and the fact that it forms the most extensive contacts with the *U5 snRNA* (65), it is tempting to speculate that Prp8 serves as a central hub, which binding to the core U5 RNPs precedes the cytoplasmic and nuclear loading of other U5-specific proteins (66). Alternatively, U5 proteins might be mutually dependent on each other, and their stable integration could be organized in a concerted fashion (62). Whether Prp8 acts as a seed that promotes binding of other U5-specific proteins is an interesting question that awaits further investigation. Interestingly, apart from the enrichment of proteins linked to the splicing machinery, SmD3 interactome revealed an overrepresentation of factors associated with ribosome biogenesis, translation, and pole cell development. These data are in accord with a report on the existence of non-canonical SmD3-containing complexes present in polar granules within the pole plasm of *Drosophila* oocytes where SmD3 guides proper localization of *oskar* mRNA likely independent of its role in splicing (67). Crosstalk between Sm proteins and ribosomes have been reported in bacteria, fly, as well as mammalian cells (68–70).

Finally, the cell deprivation of mature U5 snRNPs was associated with a broad change in gene expression and splicing profiles in *ecd* deficient larvae. Based on the enrichment of particular GO categories among differentially expressed genes, we propose that the aberrant gene expression arises as a consequence of spliceosome scarcity, which impacts transcription and pre-mRNA processing, but also reflects the adaptive stress response mounted by the different cells lacking Ecd. The upregulation and overrepresentation of genes associated with response to DNA damage and repair including *mre11* and *Ku80*, the fly ortholog of human *XRCC5*, as well as missplicing of *dre4*, a component of a conserved histone chaperone FACT complex which facilitates transcription by promoting nucleosome disassembly and assembly (71) are particularly noteworthy given the growing interest in mechanisms that link aberrant pre-mRNA processing and genome instability, including the formation of highly mutagenic DNA:RNA hybrids called R-Loops and DNA replicative stress (72–76). Whether specific transcripts are sensitive to *ecd* loss, what makes them vulnerable to missplicing, and the alleged role of Ecd in maintaining genome integrity are intriguing questions that remain to be addressed in the future.

## DATA AVAILABILITY

The raw and processed next generation sequencing and mass spectrometry proteomic data are available in the following databases:

RNA-Seq data: Gene Expression Omnibus (GEO) database (RRID:SCR_007303) GSE149056 (https://www.ncbi.nlm.nih.gov/geo/query/acc.cgi?acc=GSE149056). A link to a UCSC Genome browser session: https://genome-euro.ucsc.edu/s/dstankov/erkelenz_transcriptome_ecd1

Mass spectrometry proteomic data: ProteomeXchange (PXD) PXD019838 for Ecd protein interactome in *Drosophila* S2 cells, and PXD019761 for SmD3 protein interactome in wing imaginal discs.

## Supplementary Material

gkaa1274_Supplemental_Files
