# Supplementary material for: Ecd promotes U5 snRNP maturation and Prp8 stability
Source: Nucleic Acids Res. 2021 Jan 14;49(3):1688–707. doi: 10.1093/nar/gkaa1274 (PMC7897482; doi:10.1093/nar/gkaa1274)
Supplement: gkaa1274_Supplemental_Files [file gkaa1274_supplemental_files.zip › gkaa1274_Erkelenz et al_Supplementary Material_Figures-Tables_NAR_Proof.pdf]

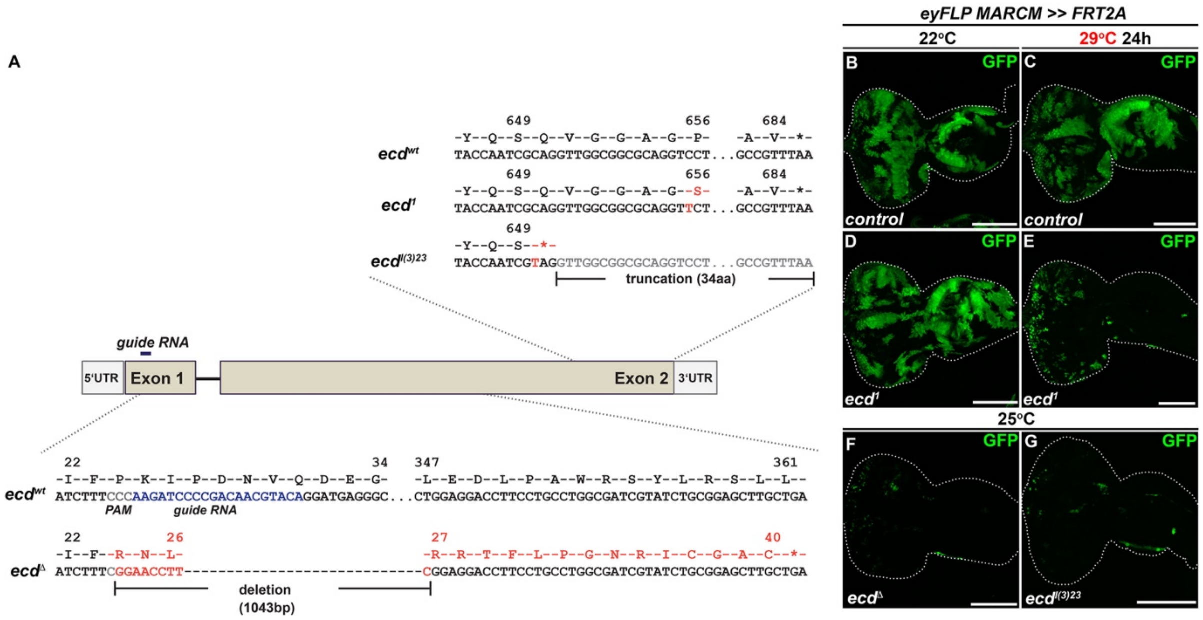

Supplementary Figure S1. Loss of *ecd* causes cell lethality.

(A) Schematic representation of the *ecd* genomic locus, mutant *ecd*^{1}, *ecd*$^{\Delta}$ and *ecd*$^{l(3)23}$ alleles and respective wild-type and mutant versions of the Ecd protein. Numbers above letters indicate amino acid (aa) positions while black and red asterisks mark endogenous and premature stop codons, respectively. In contrast to a wild-type allele (*ecd*^{wt}) encoding 684 aa long Ecd protein, the *ecd*$^{l(3)23}$ allele contains a premature stop codon due to a C to T transition, generating a truncated Ecd$^{\Delta 34}$ protein that lacks the last 34 aa at the C-terminus (light grey). Mutation of conserved proline 656 to serine (P565S) generates a conditional, temperature sensitive *ecd*^{1} allele. In the *ecd*$^{\Delta}$ allele, a frameshifting indel introduced by CRISPR-Cas9 creates a peptide comprising 40 amino acids of which only twenty-three are shared with a wild-type Ecd protein. *ecd*$^{\Delta}$ likely represents a complete loss-of-function allele. The guide RNA targeting the exon 1 is highlighted in blue, the protospacer adjacent motif (PAM) sequence is shown in dark grey, nucleotide insertions are shown in light grey, black hyphen represent deleted nucleotides. Note that the second gRNA expressed from the *pCFD5-gRNA-Ecd* construct targeting the 3‘ end of exon 2 did not cleave and was omitted from the schematic.

(B-G) Representative confocal images of third instar EADs where homozygous GFP-labelled clones of the indicated genotypes were generated using the eyFLP MARCM technique. In contrast to control clones which grow and survive at permissive (B) as well as restrictive temperature (C), majority of homozygous *ecd*^{1} clones are eliminated from EADs when larvae were upshifted to 29°C for 24 h (D, E). Only rare and extremely small *ecd*$^{\Delta}$ (F) and *ecd*$^{l(3)23}$ (G) homozygous mutant clones could be recovered in EADs of larvae grown at 25°C. Micrographs show projections of multiple confocal sections of mosaic EADs dissected from third instar larvae 7 days AEL. EAD outlines were generated based on DAPI staining. Scale bars: 100 µm.

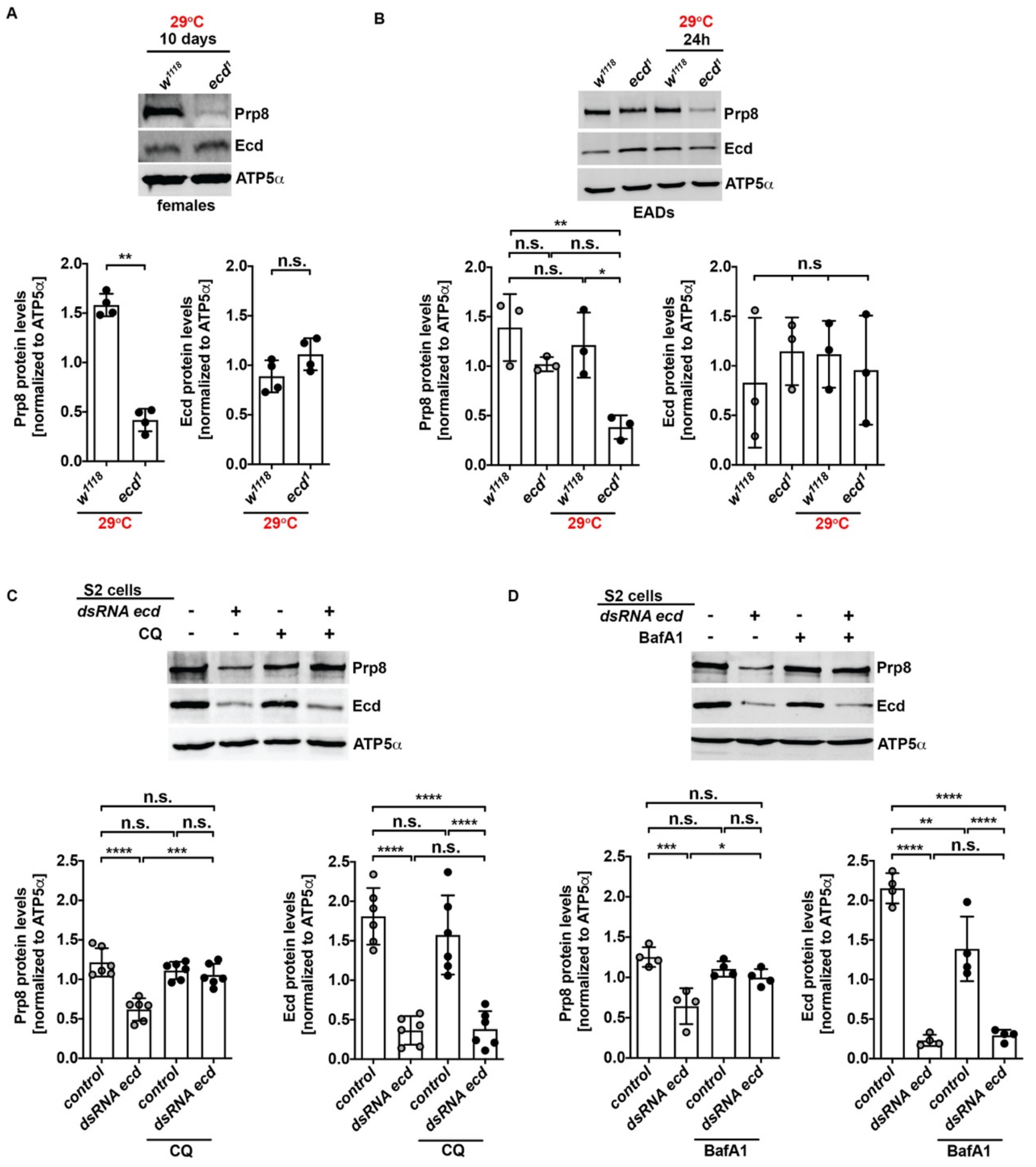

Supplementary Figure S2. ***Ecd*** **deficiency reduces Prp8 protein levels in imaginal disc cells.**

(A) Representative western blot and quantification show reduced Prp8 levels in adult female flies kept at 29°C for 10 days relative to controls (*w*^{1118}). Mitochondrial ATP synthase subunit 5 (ATP5α) served as a loading control. Data represent means ± SD, *n* = 4. Paired two-tailed Student's *t*-test was used to determine significance, $**P < 0.01$, n.s. = non-significant.

(B) Representative western blot and quantification show reduced Prp8 but not Ecd protein levels in EADs dissected from homozygous mutant *ecd*^{1} larvae kept at 29°C for 24 hours relative to controls (*w*^{1118}). Mitochondrial ATP synthase subunit 5 (ATP5α) was used as a loading control. Data represent means ± SD, *n* = 3. Two-way ANOVA with Tukey's multiple comparisons test was used to determine significance, $*P < 0.05$, $**P < 0.01$ and n.s. = non-significant.

(C-D) Prp8 levels are partially restored in *ecd*-depleted *Drosophila* S2 cells (dsRNA ecd) after blocking of the autophagy-lysosomal pathway with Chloroquine (CQ: 100 µM, 24 hours) or Bafilomycin A1 (BafA1: 400 nM, 24 hours). Control cells were treated with dsRNA against *lacZ*. Note that neither CQ nor BafA1 further stabilizes Prp8 or Ecd in control cells. Mitochondrial ATP synthase subunit 5 (ATP5α) served as a loading control. Data represent means ± SD, CQ: $n = 6$, BFA1: $n = 4$. Statistical significance was determined by ordinary one-way ANOVA with Tukey's multiple comparisons test, *$P < 0.05$, **$P < 0.01$, ***$P < 0.001$, ****$P < 0.0001$, n.s. = non-significant.

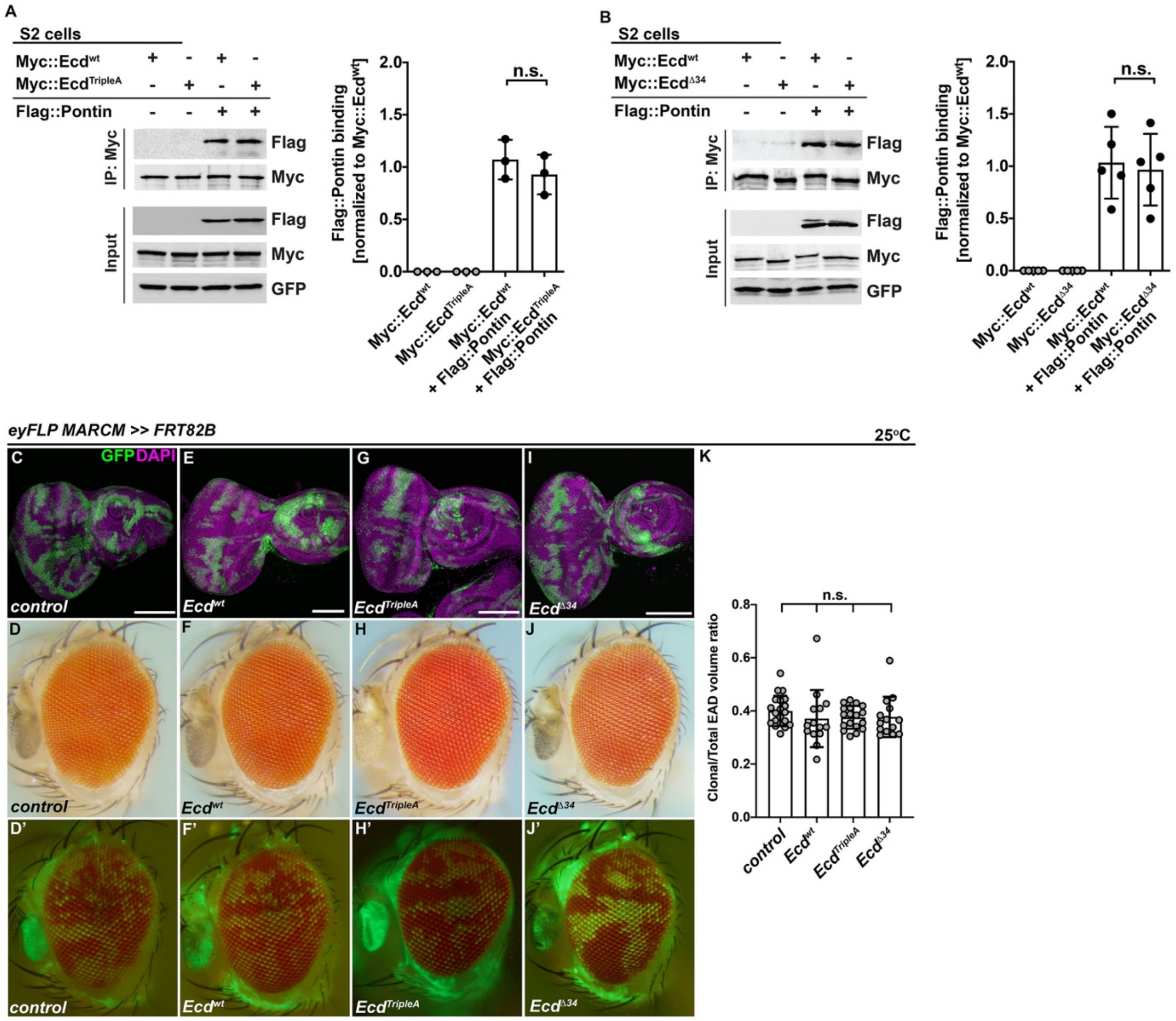

Supplementary Figure S3. Ecd^{TripleA} and Ecd$^{\Delta 34}$ mutant proteins retain capacity to interact with Pontin and their clonal overexpression induces no visible phenotypes.

(A-B) Representative western blot and quantification show that Myc-tagged wild-type Ecd, Ecd^{TripleA} (A) and truncated Ecd$^{\Delta 34}$ (B) proteins co-precipitate Flag::Pontin from *Drosophila* S2 cell lysates with the same efficiency. Myc-tagged proteins served as a bait. GFP was used as a transfection and loading control. Data represent means ± SD, n = 3. Unpaired two-tailed Student's t-test was used to determine significance, n.s. = non-significant.

(C-J) Representative confocal micrographs of mosaic third instar EADs and brightfield/fluorescent images of adult eyes where homozygous GFP-labelled clones of the indicated genotypes were generated using the eyFLP MARCM technique. Similar to GFP-labeled control EAD clones (C, D), clonal overexpression of *Ecd^{wt}* (E, F), *Ecd^{TripleA}* (G, H) or *Ecd$^{\Delta 34}$* (I, J) did not affect clone size, clone number or adult eye development. Micrographs are projections of multiple confocal sections, showing EADs 7 days AEL. Nuclei were counterstained with DAPI. Scale bars: 100 µm (C, E, G, I).

(K) Quantification of clonal to total EAD volume ratios from confocal micrographs of mosaic EADs of the indicated genotypes. Data represent means ± SD, *n* = 14-20. Ordinary one-way ANOVA with Tukey's multiple comparisons test was used to determine significance, n.s. = non-significant.

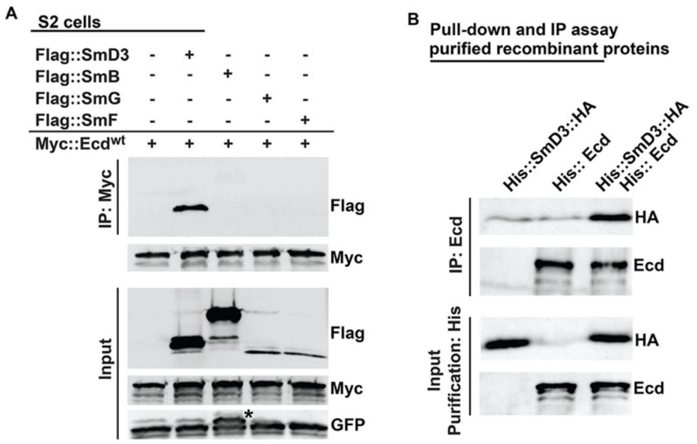

Supplementary Figure S4. Ecd directly binds SmD3, but not SmB, SmG or SmF.

(A) Co-IP assays in *Drosophila* S2 cells using the anti-Myc magnetic beads reveal a strong binding of Flag::SmD3 to Myc::Ecd^{wt} protein, compared to a weak or no interaction with Flag-tagged SmB, SmG or SmF protein. Myc::Ecd^{wt} protein served as a bait. GFP was used as a transfection and loading control. Asterisk indicates remaining signal for Flag::SmB protein detected with an anti-Flag antibody after membrane was re-probed with an anti-GFP antibody. Representative example of three independent experiments.

(B) Co-IP experiments carried out with the recombinant His-tagged SmD3::HA and Ecd proteins, which were affinity purified from bacteria, demonstrate direct binding between Ecd and SmD3. Proteins were pulled down with the Ecd antibody-coated magnetic beads. Representative example of three independent experiments.

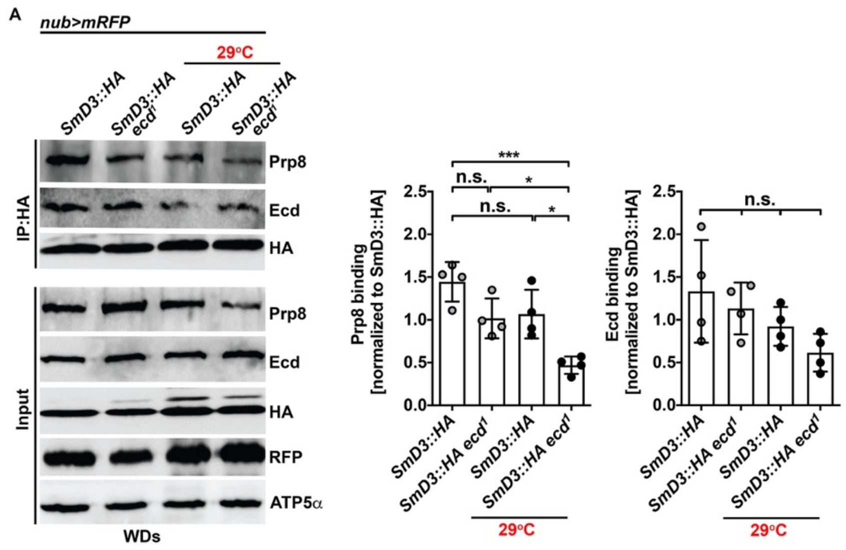

Supplementary Figure S5. Prp8 binding to SmD3 requires Ecd.

(A) Representative western blot and quantification of independent IP assays demonstrate reduced binding of endogenous Prp8 to the transgenic SmD3::HA protein in wing imaginal disc lysates of homozygous *ecd*^{1} larvae relative to control (*w*^{1118}) grown at restrictive (29°C) but not permissive temperature (18°C). Proteins were pulled down by the HA antibody-coated magnetic beads. Data represent means ± SD, *n* = 4. Statistical significance was determined by two-way ANOVA with Tukey's multiple comparisons test, **P* < 0.05, ****P* < 0.001, n.s. = non-significant.

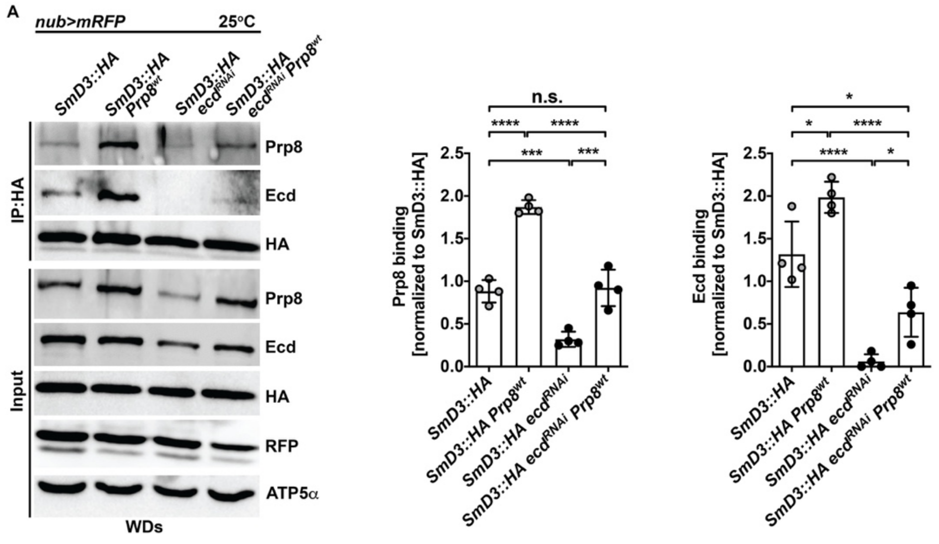

Supplementary Figure S6. Ecd and Prp8 mutually stimulate each other's binding to SmD3.

(A) Representative western blot and quantification of independent co-IPs show that overexpression of wild-type Prp8 potentiates binding of endogenous Ecd to a transgenic SmD3::HA protein in *nub>mRFP, SmD3::HA, Prp8^{wt}* wing imaginal discs compared to *nub>mRFP, SmD3::HA* samples. Note a partial recovery of *Prp8^{wt}* and SmD3::HA interaction following Prp8 overexpression in *ecd* deficient imaginal tissue. Proteins were pulled down using the HA antibody-coated magnetic beads. Data represent means ± SD, $n = 4$. Ordinary one-way ANOVA with Tukey's multiple comparisons test was used to determine significance, $^{*}P < 0.05$, $^{***}P < 0.001$, $^{****}P < 0.0001$, n.s. = non-significant.

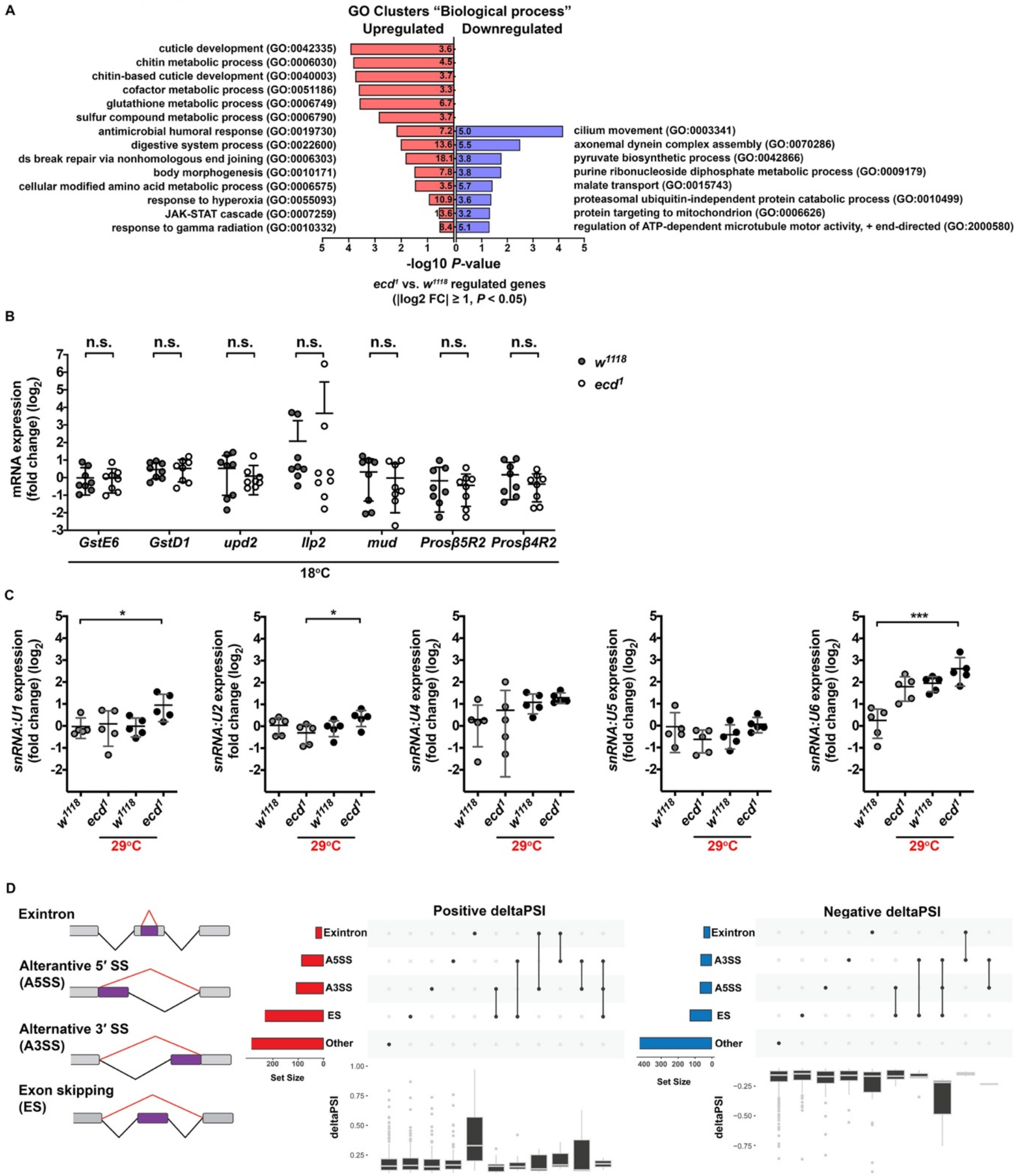

Supplementary Figure S7. ***Ecd*** **loss induces genome-wide alteration of gene expression and splicing.**

(A) The bar chart shows enriched functional GO clusters (Fold enrichment ≥ 3, $P < 0.05$, depicted within the bars, see also Supplementary Dataset S3) among genes that are up (red) and downregulated (blue) ($|\log2\ FC| \geq 1$, $P < 0.05$) in homozygous mutant *ecd*^{1} larvae relative to control (*w*^{1118}).

(B) None of the genes differentially expressed in homozygous mutant *ecd*^{1} larvae at 29°C relative to control (*w*^{1118}) (see Figure 7A, 7B and Supplementary Dataset S3) is affected at permissive temperature as determined

by RT-qPCR. Levels of *rp49* transcript were used for normalization. Data represent means ± SD, *n* = 8. Unpaired two-tailed Student's *t*-test with Welch's correction was used to determine significance, n.s. = non-significant.

(C) Levels of *U1*, *U2*, *U4*, *U4* and *U6 snRNAs* remain largely unchanged in wing imaginal discs (WDs) dissected from control (w^{1118}) and ecd^1 homozygous mutant larvae grown at permissive or restrictive temperature. RT-qPCR data are means ± SD normalized to *rp49* transcript, *n* = 5. Statistical significance was determined using two-way ANOVA with Tukey's multiple comparisons test, $^*P < 0.05$, $^{***}P < 0.001$, all other comparisons are non-significant.

(D) Classification of global alternative splicing (AS) changes in ecd^1 homozygous mutant third instar larva upshifted to a restrictive temperature for two days, including Exintron, Alternative 5'(A5SS) and 3' (A3SS) splice site usage, Exon skipping (ES) and other. Altered spliced junction usage (delta percent spliced in, deltaPSI) for each class is shown as boxplots below. See also Supplementary Dataset S4.

Supplementary Table S1. List of *Drosophila* lines

Name	*Genotype*	*Source*	*Identifier*
w^{1118}	*w*^{1118}	Bloomington *Drosophila* Stock Center	RRID: BDSC_3605
ecd^{1}	*w;; ecd*^{1}*/ TM6B*	Garen et al., 1977	
ecd$^{\Delta}$	*w;; ecd*$^{\Delta}$*/ TM6B*	this study	
ecd^{RNAi}	*w;; UAS-ecd*^{RNAi}	Claudius et al., 2014	
prp8^{RNAi}	*w; UAS-prp8*^{RNAi}	Vienna *Drosophila* Research Center (ID 18565)	
Prp8^{wt}	*w;; UAS-Prp8*^{wt}	Stankovic et al., 2020	
Ecd^{wt}	*w; pUAST-attB-Ecd*^{wt} *attP40*	this study, self-injected	
Ecd$^{\Delta34}$	*w; pUAST-Myc::Ecd*$^{\Delta34}$	this study, self-injected	
Ecd^{TripleA}	*w; pUAST-attB-Myc::Ecd*^{TripleA} *attP40*	this study, University of Cambridge Department of Genetics, Fly Facility	
SmD3::HA	*w;; UAS-SmD3::3xHA*	FlyORF Zurich (F003987)	
SmD3::HA, ecd^{RNAi}	*w;; UAS-SmD3::3xHA, UAS-ecd*^{RNAi}	this study	
SmD3::HA, Prp8^{wt}	*w;; UAS-SmD3::3xHA, pUAST-attB-Prp8*^{wt} *attP2*	this study	
SmD3::HA, ecd^{RNAi}*, Prp8*^{wt}	*w;; UAS-SmD3::3xHA, UAS-ecd*^{RNAi}*, pUAST-attB-Prp8*^{wt} *attP2*	this study	
nub>mRFP	*w; nubbin-Gal4, UAS-myr-mRFP/ CyO*	this study<br>generated from RRID:BDSC_63148 by outcrossing the P{w^{+mC}=UAS-GFP.dsRNA.R}142/TM6B, Tb^{1}	
nub>mRFP, ecd^{1}	*w; nubbin-Gal4, UAS-myr-mRFP/ CyO; ecd*^{1}*/ TM6B*	this study	
nub^{TS}*>mRFP*	*w; nubbin-Gal4, UAS-myr-mRFP/ CyO; tub-Gal80*^{TS}*/ TM6B*	this study	
nub>mRFP; SmD3::HA, Gal80^{TS}	*w; nubbin-Gal4, UAS-myr-mRFP/ CyO; UAS-SmD3::3xHA, tub-Gal80*^{TS}*/ TM6B*	this study	
ey MARCM >> FRT2A Green	*eyFLP; act>y*$^{+}$*>Gal4, UAS-GFP/* T(2;3)B3, CyO:TM6 Tb^{1}; P{w=FRT(w)}2A, *tub-Gal80*	this study	
FRT2A	w;; P{w=FRT(w)}2A	Bloomington *Drosophila* Stock Center	RRID:BDSC_1997
ecd$^{\Delta}$ *FRT2A*	*w;; ecd*$^{\Delta}$, P{w=FRT(w)}2A*/ TM6B*	this study	
Ecd^{wt}*, ecd*$^{\Delta}$ *FRT2A*	*w; pUAST-attB- Ecd*^{wt} *attP40, ecd*$^{\Delta}$, P{w=FRT(w)}2A*/ TM6B*	this study	
Ecd$^{\Delta34}$*, ecd*$^{\Delta}$ *FRT2A*	*w;; UAS-Myc::Ecd*$^{\Delta34}$*; ecd*$^{\Delta}$, P{w=FRT(w)}2A*/ TM6B*	this study	
Ecd^{TripleA}*, ecd*$^{\Delta}$ *FRT2A*	*w;; pUAST-attB-Myc::Ecd*^{TripleA} *attP40; ecd*$^{\Delta}$, P{w=FRT(w)}2A*/ TM6B*	this study	
ey MARCM >> FRT82B Green	*eyFLP; act>y*$^{+}$*>Gal4, UAS-GFP; P{ry*$^{+t7.2}$*=neoFRT}82B tub-Gal80*	Pagliarini and Xu, 2003	
FRT82B	P{ry$^{+t7.2}$=neoFRT}82B ry^{605}	Bloomington *Drosophila* Stock Center	RRID:BDSC_2035

Ecd^{wt}; FRT82B	*w; pUAST-attB- Ecd^{wt} attP40;* P{ry$^{+t7.2}$=neoFRT}82B	this study	
Ecd$^{\Delta34}$; FRT82B	*w; UAS-Myc::Ecd$^{\Delta34}$;* P{ry$^{+t7.2}$=neoFRT}82B	this study	
Ecd^{TripleA}; FRT82B	*w; pUAST-attB-Myc::Ecd^{TripleA} attP40;* P{ry$^{+t7.2}$=neoFRT}82B	this study	
nos-cas9, Lig4^{169}	*y,w, P(nos-cas9), Lig4^{169}/ FM7i, P{w^{+mC}=ActGFP}JMR3*	this study<br>generated from *y1 P(nos-cas9, w+) M(3xP3-RFP.attP)ZH-2A w** (VDRC 300001) by removing M(3xP3-RFP.attP) via Cre-mediated recombination and introducing *Lig4^{169}* from *w, Lig4^{169}* stock (RRID:BDSC_28877)	
esg^{TS}>	*w; escargot-Gal4, UAS-GFP, tub-Gal80^{TS}*	Biteau et al. 2008	
pCFD5-gRNA-Ecd	*v; pCFD5-gRNA-Ecd attP40/ CyO*	this study	
y, w, nos-phiC31; attP40	*y^{1} w* P{y[+t7.7]=nos-phiC31\int.NLS}X; P{y[+t7.7]=CaryP}attP40*	Bloomington *Drosophila* Stock Center	RRID:BDSC_79604
y, v, nos-int; attP40	*y^{1}v^{1} P{y[+t7.7]=nos-phiC31int.NLS}X; P{y[+t7.7]=CaryP}attP40*	Bloomington *Drosophila* Stock Center	RRID:BDSC_25709

Supplementary Table S2. List of oligonucleotides and plasmids

Primers used for RT-PCR

Name	*Sequence (5'-3')*	*Purpose*
prp8 For	TGGTACAAGGAGCATTGTCCACCT	qPCR
prp8 Rev	AGCCTCAACCCAATCCAGAGTTGT	qPCR
ecd For	ACCCAAGAAGCTCTACAAGCCGAA	qPCR
ecd Rev	ACCTTTGCTGTCAGTCCTGTGGAA	qPCR
rp49 For	TGTTGTCGATACCCTTGGGCTT	qPCR
rp49 Rev	TGTTGTCGATACCCTTGGGCTT	qPCR
U5 snRNA For	CTCTGGTTTCTCTTCAATTGTC	qPCR
U5 snRNA Rev	TAGACTCATTAGAGTGTTCCTC	qPCR

U2 snRNA For	CGCTTCTCGGCCTTATGGC	qPCR
U2 snRNA Rev	CTGCAATACCGGGCCAACC	qPCR
U1 snRNA For	GAGGCTTGGCCATTGCACC	qPCR
U1 snRNA Rev	AACGCCATTCCCGGCTACC	qPCR
U6 snRNA For	TCTTGCTTCGGCAGAACATATA	qPCR
U6 snRNA Rev	AAATGTGGAACGCTTCACGATT	qPCR
U4 snRNA For	CGCAGTGGCAATACCGTAAC	qPCR
U4 snRNA Rev	GGCTTCCAAAAATTGCCGTAGTGG	qPCR
GstE6 For	CCAAGGAGCGATACGATGCCA	qPCR
GstE6 Rev	CCACGAAGGCCTCAAGGGAG	qPCR
upd2 For	GAGCCTCAGCGATAGCAGCG	qPCR
upd2 Rev	ACGAAGGTCTGCATGTGGCGG	qPCR
mud For	CCTTGGAGCAGTCGGATCTGGAC	qPCR
mud Rev	GGCAGATCGACTTCCTTGTCATCCTG	qPCR
Ilp8 For	GCACAACAAGCATCACTACATCA	qPCR
Ilp8 Rev	GTTGTAGGACCTGCTCGAGTG	qPCR
GstD1 For	GTCGAGCTGAACAAGAAGCTG	qPCR
GstD1 Rev	CTCTGGTACAGCGTTCCCAT	qPCR
Prosβ5R2 For	GTGGATTCCAGAGCCACGTCGGG	qPCR
Prosβ5R2 Rev	GCAGTCGGCACTCCCTGGTC	qPCR
Prosβ4R2 For	GATCAAGGGACCAGACTTTGTGATGC	qPCR
Prosβ4R2 Rev	GCCATCATATTGAAGTCTGATAGTCGGTGG	qPCR
dre4 exon 7 For	CCACGCAACGGCAAATTCGATTC	qPCR - splicing
dre4 exon 9 Rev	CCCGGCTGTTGTACTTGTCC	qPCR - splicing

pex3 exon 1 For	TCCAAAGGCCAGCAATCGACC	qPCR - splicing
pex3 exon 2 Rev	CACTTTTTCCCGCTCCACCG	qPCR - splicing

Primers used for cloning

Name	*Sequence (5'-3')*	*Purpose*
Myc::Ecd $^{[E496A,\ E566A,\ E621A]}$ *For*	ATTGGGAATTCGGCCTGTCTAGAGAAGCTCCGCCACC	cloning into pUAST attB with EcoRI
Myc::Ecd $^{[E496A,\ E566A,\ E621A]}$ *Rev*	CACACGGTACCTCCTTCACAAAGATCCTCTAGCTTACG	cloning into pUAST attB with KpnI
$Ecd^{\Delta 34}$ *For*	AAAGTCGACATGAGCAAGATTCCAGGCAGCA	cloning into pENTR4 with SalI
$Ecd^{\Delta 34}$ *Rev*	AAAGCGGCCGCTTAGTAGCTATCCATCATGTTGC	cloning into pENTR4 with NotI
Ecd For	AAAGTCGACATGAGCAAGATTCCAGGCAGCA	cloning into pUAST attB with SalI
Ecd Rev	AAGCGGCCGCTTAAACGGCCGACTCAGATATAT	cloning into pUAST attB with NotI
PIH1D1 For	AACAGTCGACATGTCGCGTCGTTCCAATTTCATTGAG	cloning into pENTR4 with SalI
PIH1D1 Rev	AACAGCGGCCGCGAATTCTTAGTTACTGAACACTGGAATGCGAACATG	cloning into pENTR4 with NotI
Pontin For	AACAGTCGACATGAAGATCGAGGAAGTCAAGAGCACC	cloning into pENTR4 with SalI
Pontin Rev	AACAGCGGCCGCGAATTCTTACAACATAAACTTATTGTTCTTTTCGGACAAATGCTTC	cloning into pENTR4 with NotI
SmD3 For	CAACAGTCGACATGTCTATCGGAGTGCCCATTAAAGTTCTGCACGAG	cloning into pENTR4 with SalI
SmD3 Rev	AACAGCGGCCGCGACTACAGGCCGCCGCGTCCTCG	cloning into pENTR4 with NotI
SmB For	CAACGTCGACATGACGATCGGCAAGAACAACAAAATG	cloning into pENTR4 with SalI
SmB Rev	CACAGCGGCCGCTTAATAGCCACCCCTGCCCG	cloning into pENTR4 with NotI
SmG For	CAACGTCGACATGTCGAAGGCCCATCCCC	cloning into pENTR4 with SalI
SmG Rev	CACAGCGGCCGCCTAGACCCTGTCCAGGGCC	cloning into pENTR4 with NotI
SmF For	CAAAGGATCCGGATGTCGGCTGGTATGCCCATT	cloning into pENTR4 with BamHI
SmF Rev	CACAGCGGCCGCCTAGTCGCGCATTTCGCCC	cloning into pENTR4 with NotI
SmD3::1xHA For	TTCTGCGCGCACAGGCTCGTGGCAGAGGAAGAGG	cloning into pENTR4-SmD3 with BssHII
SmD3::1xHA Rev	TAGTCGCGGCCGCTTACGCATAGTCAGGAACATCGTATGGGTAGCCCAGGCCGCCGCG	cloning into pENTR4-SmD3 with NotI

pCFD5_gEcd Fwd	GCGGCCCGGGTTCGATTCCCGGCCGATGCA<br>TGTACGTTGTCGGGGATCTT*GTTTTAGAGCT*<br>*AGAAATAGCAAG*	tRNA seq,<br>*gRNA core*<br>pCFD5 cloning
pCFD5_gEcd Rev	ATTTTAACTTGCTATTTCTAGCTCTAAAACGT<br>AATCCCGCTTAAACGGCC*TGCACCAGCCGG*<br>*GAATCGAACCC*	tRNA seq,<br>*gRNA core*<br>pCFD5 cloning
dEcd 3-UTR CRISPR screen Rev	GGAGCTGGCTACCACAACAACCAGGA	screening CRISPR-Cas9 deletion
dEcd 5-UTR CRISPR screen For	ACTAGCTGGAGTGTCAAGTGGA	screening CRISPR-Cas9 deletion

Primers used for *in vitro* transcription

Name	*Sequence (5'-3')*
LacZ For	TAATACGACTCACTATAGGTAAATTGTAAGCGTTAATATTTTG
LacZ Rev	TAATACGACTCACTATAGGAATTCGATATCAAGCTTATCGAT
ecd 3'UTR For	TAATACGACTCACTATAGGGGCGGGATTAC AGGAATTAGT ATAGTC
ecd 3'UTR Rev	TAATACGACTCACTATAGGGACATTGAAATATTCACATTGACGTTTATTTAATGG

Plasmids

Name	*Purpose*
pENTR4 Ecd^{wt}	Gateway entry vector, cloning
pENTR4 Ecd$^{\Delta 34}$	Gateway entry vector, cloning
pENTR4 Ecd$^{[S496A,E566A,E621A]}$	Gateway entry vector, cloning
pENTR4 PIH1D1	Gateway entry vector, cloning
pENTR4 Pontin	Gateway entry vector, cloning
pENTR4 SmD3	Gateway entry vector, cloning
pENTR4 SmB	Gateway entry vector, cloning
pENTR4 SmG	Gateway entry vector, cloning
pENTR4 SmF	Gateway entry vector, cloning
pENTR4 SmD3::1xHA	Gateway entry vector, cloning
pTMW Ecd^{wt}	S2 cell expression, Co-IP

pTMW Ecd$^{\Delta 34}$	S2 cell expression, Co-IP, *Drosophila* transgenesis
pTMW Ecd$^{[S496A,E566A,E621A]}$	S2 cell expression, Co-IP
pTFW PIH1D1	S2 cell expression, Co-IP
pTFW Pontin	S2 cell expression, Co-IP
pTFW SmD3	S2 cell expression, Co-IP
pTFW SmB	S2 cell expression, Co-IP
pTFW SmG	S2 cell expression, Co-IP
pTFW SmF	S2 cell expression, Co-IP
pDEST17-SmD3::1xHA	*E.coli BL21* expression, Co-IP
pET28b Ecd	*E.coli BL21* expression, Co-IP
pUAST attB Myc::Ecd^{wt}	*Drosophila* transgenesis
pUAST attB Myc::Ecd $^{[E496A, E566A, E621A]}$	*Drosophila* transgenesis
